# Chronic TCR-MHC (self)-interactions limit the functional potential of TCR affinity-increased CD8 T lymphocytes

**DOI:** 10.1186/s40425-019-0773-z

**Published:** 2019-11-05

**Authors:** Minh Ngoc Duong, Efe Erdes, Michael Hebeisen, Nathalie Rufer

**Affiliations:** Department of oncology UNIL CHUV, Lausanne University Hospital and University of Lausanne, CH-1066 Epalinges, Switzerland

**Keywords:** Immunotherapy, Preclinical study, CD8 T cells, NY-ESO-1 tumor antigen, TCR affinity optimization, TCR/CD3 complex, T cell activation, Receptor signaling, T cell function

## Abstract

**Background:**

Affinity-optimized T cell receptor (TCR)-engineered lymphocytes targeting tumor antigens can mediate potent antitumor responses in cancer patients, but also bear substantial risks for off-target toxicities. Most preclinical studies have focused on T cell responses to antigen-specific stimulation. In contrast, little is known on the regulation of T cell responsiveness through continuous TCR triggering and consequent tonic signaling. Here, we addressed the question whether increasing the TCR affinity can lead to chronic interactions occurring directly between TCRs and MHC-(self) molecules, which may modulate the overall functional potency of tumor-redirected CD8 T cells. For this purpose, we developed two complementary human CD8 T cell models (i.e. HLA-A2 knock-in and knock-out) engineered with incremental-affinity TCRs to the HLA-A2/NY-ESO-1 tumor antigen.

**Methods:**

The impact of HLA-A2 recognition, depending on TCR affinity, was assessed at the levels of the TCR/CD3 complex, regulatory receptors, and signaling, under steady-state conditions and in kinetic studies. The quality of CD8 T cell responses was further evaluated by gene expression and multiplex cytokine profiling, as well as real-time quantitative cell killing, combined with co-culture assays.

**Results:**

We found that HLA-A2 per se (in absence of cognate peptide) can trigger chronic activation followed by a tolerance-like state of tumor-redirected CD8 T cells with increased-affinity TCRs. HLA-A2^pos^ but not HLA-A2^neg^ T cells displayed an activation phenotype, associated with enhanced upregulation of c-CBL and multiple inhibitory receptors. T cell activation preceded TCR/CD3 downmodulation, impaired TCR signaling and functional hyporesponsiveness. This stepwise activation-to-hyporesponsive state was dependent on TCR affinity and already detectable at the upper end of the physiological affinity range (K_D_ ≤ 1 μM). Similar findings were made when affinity-increased HLA-A2^neg^ CD8 T cells were chronically exposed to HLA-A2^pos^-expressing target cells.

**Conclusions:**

Our observations indicate that sustained interactions between affinity-increased TCR and self-MHC can directly adjust the functional potential of T cells, even in the absence of antigen-specific stimulation. The observed tolerance-like state depends on TCR affinity and has therefore potential implications for the design of affinity-improved TCRs for adoptive T cell therapy, as several engineered TCRs currently used in clinical trials share similar affinity properties.

## Background

Recent progress in oncology has shown that cytotoxic CD8 T cells are essential players in generating protective and durable immune responses against cancer. Efficient triggering of T cell responses is mainly dictated by the strength of T cell receptor (TCR) binding to cognate peptide-MHC (pMHC), i.e. the TCR-pMHC affinity/avidity. Seminal clinical trials demonstrated the importance of TCR-pMHC affinity/avidity in cancer patients treated with engineered T cells of enhanced TCR affinity [1]. Clinical studies performed with affinity-enhanced T cells against the cancer testis HLA-A2/NY-ESO-1_157-165_ antigen provided augmented in vivo functional capacity and improved tumor growth control [2–4]. The genetically modified TCR (1G4 α95:LY) has been largely used to treat patients with melanoma, sarcoma or multiple myeloma without major harmful effects [2–4]. In contrast, the clinical success of affinity-enhanced TCRs specific for other antigens was associated with off-target adverse events, leading to serious and potentially lethal toxicities [1], as with the MAGE-A3/HLA-A1 TCR [5, 6]. Compiled data from numerous experimental models further indicate that T cell activation and subsequent function can be limited to a particular TCR-pMHC affinity window [7]. Notably, T cells expressing TCRs with increased affinity, above the physiological range, or with prolonged half-lives, display substantial functional defects. This likely involves the presence of negative feedback mechanisms, which may prevent overreactive T cell responses [8, 9]. Collectively, there is a clear need to promote better preclinical strategies, including the prediction of optimized T cell responsiveness and off-target toxicity related to enhanced TCR-pMHC affinity, to guarantee the safety of candidate TCRs for clinical testing.

While tolerance mechanisms related to TCR affinity have been well documented in thymocytes during central tolerance induction [10], only limited information is available on the regulatory processes underlying peripheral T cell-mediated responses against tumor or microbial antigens according to TCR affinity. Models using affinity-matured TCR variants [11] or altered peptide ligands [12] have revealed defined tolerogenic mechanisms such as deletion or anergy. For instance, CD8 T cells engineered with a TCR of nanomolar affinity are rapidly deleted through mechanisms of peripheral T cell tolerance [11]. Moreover, increasing the TCR signaling strength by altered peptide ligands favors anergy induction [12]. Gallergos et al. further described that CD4 T cells expressing a TCR of higher avidity were less able to control *Mycobacterium tuberculosis* infection in vivo than T cells of intermediate avidity [13]. Specifically, this study identified programmed TCR downregulation as a potential mechanism restricting high avidity CD4 T cell responses at the peak of clonal expansion [13]. Along this line, we reported that SHP-1 phosphatase activity and PD-1 were involved in limiting T cell signaling and function, depending on TCR affinity, in tumor-specific CD8 T cells of increased-affinity TCRs [9, 14]. Together, these observations revealed the presence of negative feedback mechanisms restricting antigen-specific T cell responses in relation to the TCR-pMHC affinity.

TCR affinity-optimization strategies imply the modification of TCR sequences by inserting point-mutations within the complementary-determining regions (CDRs) of the TCRα- and/or β-chains. Initial studies showed that high affinity TCR variants generated by mutations in the CDR1, CDR2 or CDR3 loops retained remarkable peptide specificity [15]. Single and dual CDR3α and CDR2β amino acid changes further allowed the enhancement of antigen-specific reactivity in TCR-redirected CD4 and CD8 T cells [16]. Through a rational design approach, we previously established a panel of incremental affinity to the HLA-A2/NY-ESO-1 tumor antigen, mostly involving amino-acid changes in CDR2β combined to single point-mutations within CDR3β and/or CDR2α [9, 17]. These TCR affinity-enhanced variants retained NY-ESO-1 specificity and similar peptide recognition patterns as the wild-type receptor [17]. Since improved TCR affinity (K_D_ ≤ 1 μM) mainly resulted from increased contacts with the HLA-A2 (referred to as A2) backbone [17], we hypothesized that A2-(self) molecules per se may directly trigger chronic interactions with affinity-increased TCRs and modulate the functional state of tumor-redirected CD8 T cells, even in the absence of cognate peptide. To address this issue, we generated two complementary CD8 T cell models. Jurkat J76 CD8αβ T cells (A2 knock-in) engineered with affinity-increased TCRs were used to assess the impact of A2 at the TCR/CD3 complex, regulatory receptor and signaling levels, under steady-state conditions and in kinetic studies. TCR-redirected primary CD8 T lymphocytes, knocked-out for the A2 allele (i.e. A2^neg^) or not (A2^pos^), further provided a unique experimental setting for evaluating the quality of T cell responses through various biological outcomes. Together, our data provide strong evidence that chronic TCR-A2 (self)-interactions can directly induce the early activation of tumor-redirected CD8 T cells, followed by a tolerance-like state. Importantly, this occurred readily in T cells expressing TCRs at the upper limit of the natural affinity range, indicating possible consequences for T cell adoptive immunotherapy, currently based on such TCR affinity-optimization strategies [1].

## Materials and methods

### Culture of cell lines and primary CD8 T lymphocytes

HLA-A2^neg^/J76 CD8αβ cells (kindly provided by Drs. I. Edes and W. Uckert; Max-Delbrück-Center, Berlin, Germany, *unpublished data*), HLA-A2^pos^/TAP-deficient T2 cells (ATCC CRL-1992), HLA-A2^pos^/NY-ESO-1^neg^ NA8 cells (CVCL-S599) were cultivated and primary CD8 T lymphocytes were generated from peripheral blood cells as described in detail in the Additional file 1.

### Generation of CRISPR-A2 primary CD8 T cells and CRISPR-A2 NA8 tumor cells

CRISPR-A2 primary CD8 T cells and NA8 cells were generated based on the design of the 20 nucleotide-single guide (sgRNA) sequence targeting HLA-A*0201 (GAGGGTCCGGAGTA TTGGGA) as described in detail in the Additional file 1. In brief, following the generation of the lenti-CRISPR-A2 plasmid and subsequent production of lentiviral particles, ultra-concentrated supernatant was used to infect freshly isolated A2^pos^ CD8 T lymphocytes after 24 h stimulation with CD3/CD28 beads (1st expansion) or NA8 cells to create CRISPR-A2 (A2^neg^)-CD8 T cells or CRISPR-A2 (A2^neg^)-NA8 cells, respectively. Lenti-CRISPR-EGFP sgRNA 6 (Addgen plasmid #51765) was used as a mock control. Transduced cells (A2^neg^) were sorted to purity with PE-labeled HLA-A2 antibody by flow cytometry (FACSAriaII, BD Biosciences).

### Generation of A2^pos^ J76 CD8αβ T cells

HLA-A*0201 sequence was codon-optimized with Geneart tool (Thermofisher) and subsequently cloned into pRRL lentiviral plasmid. The sequence was confirmed by DNA sequencing. Supernatant of lentiviral-transfected 293 T cells was used to infect A2^neg^ J76 CD8αβ cells, allowing the generation of A2^pos^ J76 CD8αβ cells. Surface expression of A2 molecules was assessed with PE-labeled HLA-A2 antibody by flow cytometry (FACSAriaII, BD Biosciences) and yielded over 95% of A2^pos^ J76 CD8αβ cells.

### Generation of TCR-engineered primary CD8 T cells and J76 CD8αβ T cells

The plasmids encoding for the panel of incremental affinity TCRs against A2/NY-ESO-1_157-165_ (Additional file 1: Table S1) were cloned as described in the Additional file 1. Supernatant of lentiviral-transfected 293 T cells was used to infect (i) A2^pos^ and A2^neg^ primary CD8 T cells stimulated for 24 h with CD3/CD28 beads (1st expansion), (ii) CRISPR-A2 and CRISPR-EGFP primary CD8 T cells stimulated for 24 h with phytohemagglutinin (PHA) and A2^neg^ feeder cells (2nd expansion), or (iii) A2^pos^ and A2^neg^ J76 CD8αβ T cells. For functional analysis, primary CD8 T cells were sorted between 15 and 21 days post TCR transduction with PE-labeled A2/NY-ESO-1_157-165_-specific multimer by flow cytometry (FACSAriaII, BD Biosciences). During 10 days after re-stimulation with PHA/A2^neg^ feeder cells (3rd expansion), primary CD8 T cells were regularly counted by trypan blue and population doubling was calculated based on the initial cell number obtained after sort with multimer.

### Surface staining by flow cytometry

Surface staining was performed by incubating 1-3 × 10^5^ TCR-transduced A2^pos^ and A2^neg^ J76 CD8αβ cells or primary CD8 T cells at 4 °C with NY-ESO-1 multimers for 40 min and/or corresponding antibodies (panTCRαβ, CD3ε, CD5, PD1, TIM-3, TIGIT, 2B4, CD69, CD25, 4-1BB, CD28; Additional file 1: Table S2) for 20 min. For total CD3ε expression analysis, cells were fixed in PBS 1% formaldehyde before being stained with the corresponding antibody and permeabilized with 0.1% saponin. Annexin V and Ki67 staining were performed according to the manufacturer’s instructions (BD Biosciences). All experiments were performed under unstimulated, resting culture conditions. Samples were acquired with a Gallios (Beckman Coulter) flow cytometer and data were analyzed by FlowJo software (Tree star, v10.0.8). Co-expression analysis was performed with SPICE software (v.5.35, NIH, Bethesda).

### Phospho-flow assay

2.5 × 10^5^ TCR-transduced A2^pos^ and A2^neg^ J76 CD8αβ cells were left unstimulated or stimulated with either 1 μg/ml unlabeled A2/NY-ESO-1_157-165_ multimer or 10 μg/ml OKT3 anti-CD3ε antibody or 1 μg/ml PMA and 250 ng/ml ionomycin for 5 min. Cells were fixed with 4% paraformaldehyde (Polysciences) for 10 min at 37 °C followed by permeabilization with 100% ice-cold methanol (Sigma Aldrich) for 20 min before being stained with the following antibodies for 30 min at room temperature: anti-phospho-CD3ζ (CD247) Alexa Fluor647 (Y142, Clone: K25–407.6, BD Phosflow), anti-phospho-ERK1/2 Alexa Fluor® 647 (T202/Y204 of ERK1 and T185/Y187 of ERK2, Clone: E10, Cell Signaling Technology), and anti-total c-CBL (Clone: YE323**,** Abcam). Samples were acquired with a Gallios (Beckman Coulter) flow cytometer and data were analyzed by FlowJo software (Tree star).

### Microarray analysis

Genome-wide microarray analysis was previously performed on A2^pos^ primary CD8 T cells engineered with the panel of NY-ESO-1-specific TCR variants (GSE42922) [9]. Gene set enrichment was analyzed with GSEA (www.broadinstitute.org/gsea). Enrichment was considered significant if nominal *p* value was < 0.05 and false discovery rate (FDR) was < 0.25.

### Multiplex cytokine profiling assay

0.1 × 10^6^ A2^pos^ or A2^neg^ primary CD8 T cells were seeded in 96-well plate. After 1 day, cells were left either unstimulated or were stimulated with 0.1 nM (0.025 μg/ml) unlabeled A2/NY-ESO-1_157-165_ multimer for 24 h. Supernatants were harvested and cytokine concentrations were measured with bead-based LEGENDplexTM human Th cytokine panel (Biolegend) according to the manufacturer’s instructions.

### Real-time IncuCyte killing assay

10^4^ A2^pos^ NA8 cells were seeded per well in 96-well plates 1 day before TCR-transduced A2^pos^ (CRISPR/GFP) or A2^neg^ (CRISPR/A2) primary CD8 T cells were added at an E:T ratio of 1:10, with or without 1 nM NY-ESO-1_157-165_ peptide. IncuCyte caspase-3/7 reagent (Essen Bioscience) was added at 10 μM final. Cell confluence and apoptosis were monitored every 2 h for 3–4 days with 10X camera in the IncuCyte system. Data was analyzed with the integrated software.

### Coculture experiments

For TCR-redirected primary CD8 T cell cocultured with NA8 cells, 3 × 10^5^ A2^pos^ or A2^neg^ NA8 cells were seeded per well in 24-well plates for 1 day, and CRISPR-A2 (A2^neg^) CD8 T cells expressing the indicated TCR variants were added at a 1:1 ratio and cocultured for 3 days. For long-term cocultures, A2^neg^ CD8 T cells were passed every 3–4 days on a new layer of A2^pos^ or A2^neg^ NA8 cells. Cells were harvested and labeled with antibodies before being analyzed by flow cytometry. CD8 T cells and NA8 cells were distinguished based on FCS-SSC parameters and CD8 staining. In the mixed primary CD8 T cell coculture, CRISPR-A2 (A2^neg^)- and CRISPR-EGFP (A2^pos^) CD8 T cells were mixed at a 1:1 ratio immediately prior to TCR transduction by lentivectors. Cells were stained with surface antibodies and analyzed by flow cytometry at indicated time post-TCR transduction. A2^pos^ and A2^neg^ CD8 T cells were distinguished based on the A2 staining.

### Statistical analysis

Data were analyzed using Prism software (GraphPad, v.7.03). Direct comparison between A2^pos^ (CRISPR/GFP) and A2^neg^ (CRISPR/A2) primary CD8 T cells or A2^pos^ and A2^neg^ J76 CD8αβ T cells transduced with the same TCR variant were performed using matched, two-way ANOVA followed by Sidak’s multiple comparisons test. The numbers of independent experiments as well as the associated *P* values at α = 0.05 wherever the difference was significant are indicated throughout the manuscript.

## Results

### Downregulation of basal TCR/CD3 complex depends on both TCR affinity and HLA-A2 expression

**Fig. 1:**
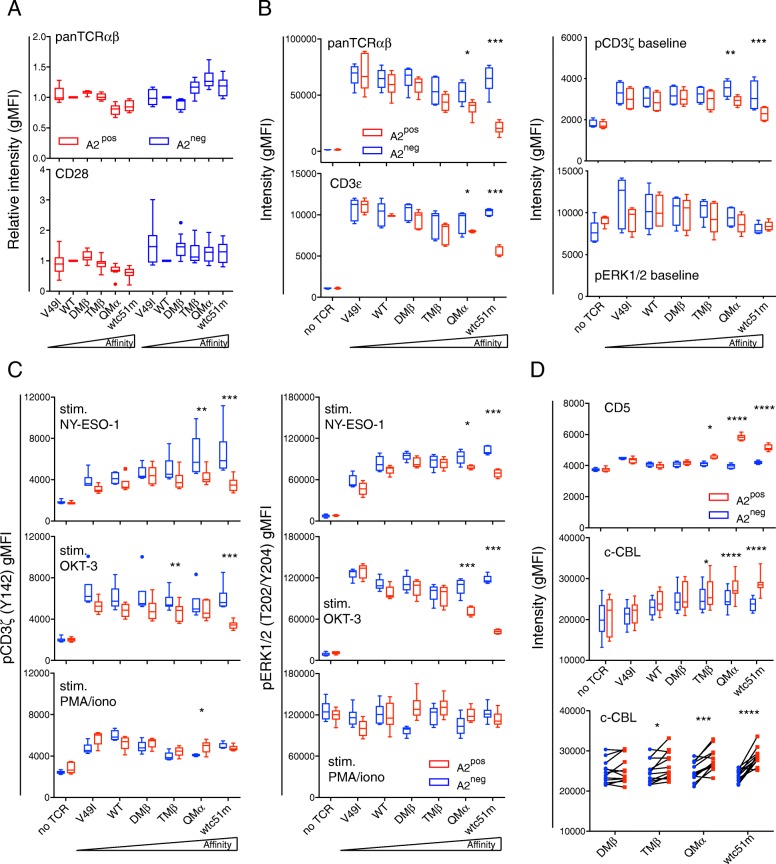
Baseline expression levels of the TCR/CD3 complex, CD5 and c-CBL and phosphorylation levels of CD3ζ and ERK upon stimulation in relation to HLA-A2. **a, b** Expression levels of TCR/CD3 complex and CD28 in A2^pos^ and A2^neg^ primary CD8 T cells (**a**) or A2^pos^ and A2^neg^ J76 CD8αβ cells (**b**) engineered with TCRs of incremental affinities and analyzed under steady-state culture condition, in the absence of cognate antigen. **c** Quantification of CD3ζ and ERK1/2 phosphorylation in TCR-expressing A2^pos^ and A2^neg^ CD8αβ J76 cells after stimulation with NY-ESO-1 multimer, OKT3 (anti-CD3ε) antibody or PMA/Ionomycin. **d** Quantification of CD5 and c-CBL expression in A2^pos^ and A2^neg^ CD8αβ J76 cells. Direct comparison (bottom panel) of c-CBL expression in A2^neg^ versus A2^pos^ CD8αβ J76 cells for the indicated TCR variants by two-tailed, paired *t* test. **a-d** Data are means ± SD and representative of 5 to 15 independent experiments. TCR-transduced A2^pos^ cells versus A2^neg^ T cells are depicted as red versus blue symbols. * *P* ≤ 0.05, ** *P* ≤ 0.01, *** *P* ≤ 0.001 and **** *P* ≤ 0.0001

Through structure-based rational predictions [18], we have established a panel of affinity-increased TCRs for the NY-ESO-1 tumor antigen presented in the context of HLA-A2 (referred to as A2) (Additional file 1: Table S1 [17];), and reported significant reduced levels of surface TCR/CD3ε in A2^pos^ primary CD8 T cells redirected with increased affinity TCRs [14]. This was readily observed under steady-state conditions, i.e. in long-term cultures of primary resting CD8 T cells in the absence of cognate antigen, that were periodically restimulated with phytohemagglutinin and feeder cells. Extending on this study, however, we did not find major changes in the baseline expression of TCRαβ or of CD28 costimulatory receptor within engineered CD8 T lymphocytes expressing the same TCR affinity panel but lacking A2 (Fig. 1 a). These data suggest a critical role of A2 expression in modulating basal levels of the TCR/CD3 complex, according to TCR affinity. Therefore, we next used the Jurkat 76 (i.e. J76) A2^neg^ T cell subline, which is devoid of endogenous TCRαβ chains, but was modified to express CD8αβ (Edes and Uckert et al., *unpublished data*; Additional file 1: Figure S1A), to study the function of the redirected TCR complex and its components. We generated J76 CD8αβ T cells expressing A2 de novo by lentiviral transduction and showed that the A2 molecules were fully functional (Additional file 1: Figure S1B, C). Following TCR transduction with affinity-enhanced variants and under resting culture conditions, levels of surface TCRαβ and CD3ε as well as of the proximal phospho-CD3ζ signaling molecule were down-modulated in de novo A2-expressing J76 cells along the TCR affinity gradient, when compared to A2^neg^ J76 cells (Fig. 1 b, Additional file 1: Figure S1D). This was not the case for the total (i.e. intracellular and extracellular) levels of CD3ε (Additional file 1: Figure S1E) and the distal ERK1/2 signaling node (Fig. 1 b). Together, these results demonstrate that basal downregulation of the TCR/CD3 complex and associated CD3ζ in redirected J76 CD8αβ T cells depends on both TCR affinity and A2 expression.

### Basal TCR/CD3 downregulation leads to impaired TCR signaling capacity

To assess whether this TCR/CD3 downregulation state impacts on the signaling potential of the J76 CD8αβ T cells in response to cell activation, we measured the phosphorylation levels of CD3ζ and ERK1/2 under various stimulation conditions (Fig. 1 c, Additional file 1: Figure S2A). Antigen-specific TCR-triggering by A2/NY-ESO-1_157-165_ multimers and TCR-dependent cross-linking with the CD3ε/OKT3 antibody generated drastic declines in both phospho-CD3ζ and phospho-ERK1/2 levels. This mostly occurred for the TCR variants displaying TCR affinities at the limit or above the natural affinity range (i.e. TMβ, QMα and wtc51m) and was only detected in A2^pos^ J76 cells. In contrast, stimulation with PMA/ionomycin, which bypasses the TCR/CD3 complex, led to comparable patterns of CD3ζ and of ERK1/2 phosphorylation between A2^pos^ and A2^neg^ J76 CD8αβ T cells and across the TCR affinity range (Fig. 1 c). This indicates that basal TCR/CD3 downregulation according to TCR affinity leads to reduced proximal (CD3ζ) and distal (ERK1/2) signaling capacity after TCR-mediated cell activation. Again, impairment in TCR signaling required the presence of A2.

### Basal TCR/CD3 downregulation is linked to enhanced CD5 and c-CBL levels

TCR signaling is negatively regulated by the E3 ubiquitin-protein ligase c-CBL and the CD5 co-receptor. Whereas c-CBL has been shown to control ubiquitination and degradation of the CD3 chains [19], CD5 acts as a scaffold for c-CBL-mediated ubiquitylation in response to TCR stimulation [20, 21]. Given the importance of both molecules in TCR signaling regulation, we wondered whether CD5 and c-CBL were involved in the TCR/CD3 downregulation state observed in A2-expressing J76 T cells of high affinity TCRs. Under baseline conditions, we found significant increased expression of CD5 and of total c-CBL in A2^pos^ J76 CD8αβ T cells with high affinity TCRs (Fig. 1 d, Additional file 1: Figure S2B). Conversely, only minimal differences in expression were seen in J76 cells lacking the A2 molecule. These observations suggest that downmodulation of the TCR/CD3 complex could be mediated by CD5 and c-CBL, whereby c-CBL might directly be involved in CD3ζ degradation through ubiquitination processes. Once more, this was already observed in engineered CD8 T cells expressing TCRs at the upper end of the physiological affinity range (i.e. TMβ) and occurred through the recognition of A2.

### Phenotypic activation precedes TCR/CD3 downregulation upon affinity-increased TCR transduction

**Fig. 2:**
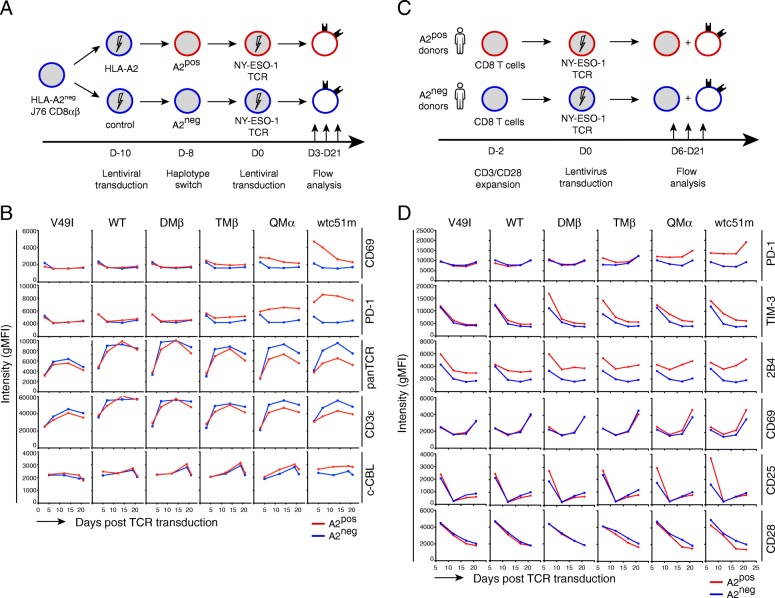
Kinetics of the expression of co-activating/inhibitory surface receptors upon affinity-increased TCR transduction in the absence of cognate peptide antigen. **a, c** Schematic representations of the phenotype characterization of A2^pos^ and A2^neg^ J76 CD8αβ cells (**a**) or A2^pos^ and A2^neg^ primary CD8 T cells from individual healthy donors (**c**) following affinity-increased TCR transduction, in the absence of antigen-specific stimulation. **b and d** Kinetic analyses of CD69, PD-1, TCR/CD3ε complex and c-CBL in redirected J76 CD8αβ cells (**b**) or of co-activating/co-inhibitory receptor expression in redirected primary CD8 T cells (**d**) from day 3 or 6 up to day 21 after TCR transduction. Data are representative of 2 to 4 independent experiments

TCR downmodulation was previously reported as a consequence of T cell activation [13, 22]. Hence, we next explored the dynamics of TCR/CD3ε downmodulation alongside to co-activating/co-inhibitory receptor expression, by performing kinetic studies, in which A2^pos^ and A2^neg^ J76 CD8αβ T cells were followed for various time-points upon TCR transduction (Fig. 2 a). Rapid (at day 3) and sustained expression of the activation marker CD69 associated to increased PD-1 levels were exclusively found in TCR affinity-increased A2^pos^ J76 cells (Fig. 2 b, Additional file 1: Figure S2C). From day 7 and onwards, these cells, in contrast to A2^neg^ cells, also displayed reduced surface expression of TCRαβ and of CD3ε, which inversely correlated to augmented total CBL protein. Similar longitudinal experiments were performed on primary CD8 T lymphocytes isolated from A2^pos^ and A2^neg^ healthy donors following TCR transduction (Fig. 2 c). Consistent with the data obtained for the J76 model, several co-inhibitory receptors (i.e. PD-1, TIM-3, 2B4) and activation markers (i.e. CD25, CD69) were found rapidly upregulated in A2^pos^ but not A2^neg^ primary T cells, according to TCR affinity (Fig. 2 d). Except for CD25, this upregulation was maintained over-time post-TCR transduction, and contrasted to the downmodulation of CD28 costimulatory molecule occurring only at later time-points (Fig. 2 d) or to the reduced TCRαβ levels found in long-term cultures (Fig. 1 a [14];). Overall, these data revealed a step-wise early activation state followed by TCR/CD3 and CD28 downregulation in A2^pos^ CD8 T cells of increased affinity TCRs.

### A2 expression is required to induce strong activation upon affinity-increased TCR transduction in primary CD8 T cells

**Fig. 3:**
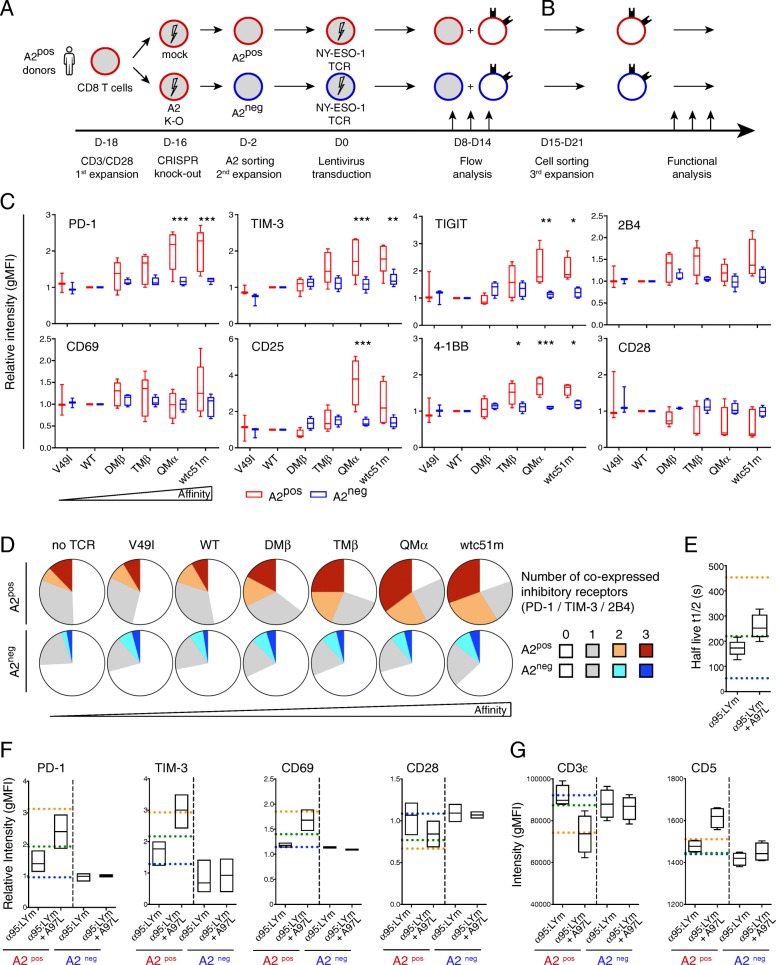
Basal expression of co-activating/inhibitory receptors upon affinity-increased TCR transduction in relation to HLA-A2. **a** Schematic representation of the experimental design using A2-KO CD8 T cells. A2^pos^ primary CD8 T cells were first knocked-out for HLA-A2 by CRISPR/Cas9, expanded by PHA/A2^neg^ feeder cells and transduced with affinity-increased TCRs, before being characterized by flow cytometry (from day 8 to day 14). **b** For functional analyses, TCR-redirected A2^pos^ and A2^neg^ primary CD8 T cells were further purified by FACS-sorting (between D15–21) and expanded using PHA/A2^neg^ feeder cells. **c** Quantification of the expression of co-activating/inhibitory receptors on A2^pos^ (CRISPR/GFP) and A2^neg^ (CRISPR/A2) primary CD8 T cells from day 8 to day 14 post-TCR transduction, independently of antigen-specific stimulation. Data are depicted as means ± SD (relative to the WT TCR variant) and representative of 4 to 5 independent experiments. * *P* ≤ 0.05, ** *P* ≤ 0.01, and *** *P* ≤ 0.001. **d** Co-expression of 0 to 3 co-inhibitory (PD-1, TIM-3 and 2B4) receptors of A2^pos^ (CRISPR/GFP) and A2^neg^ (CRISPR/A2) primary CD8 T cells. **e** TCR-pMHC off-rate measurements of two CDR3-based TCR variants (α95:LYm and α95:LYm/A97L). **f** Quantification of PD-1, TIM-3, CD69 and CD28 expression levels in primary CD8 T cells redirected with CDR3-based TCR variants, in the absence of antigen-specific stimulation. **g** Expression levels of TCR/CD3 complex and of CD5 in redirected J76 CD8αβ cells with CDR3-based TCR variants, analyzed under steady-state culture conditions. **e-g** Data are depicted in comparison to WT (blue dotted line), DMβ (green line) and TMβ (orange line) TCR variants and are representative of 2 to 4 independent experiments

To determine whether A2 expression has a direct impact on the activation status of affinity-improved CD8 T cells, we generated A2 knock-out primary CD8 T cells with a sequence-specific CRISPR/Cas9 lentiviral construct. A2^pos^ (i.e. CRISPR/mock) and A2^neg^ (i.e. CRISPR/A2) CD8 T cells sharing the same cellular background were then transduced with the affinity-increased TCR panel, expanded non-specifically with PHA/A2^neg^-feeder cells and characterized for their expression of co-activating/co-inhibitory receptors (Fig. 3 a). From day 8 to 14 following TCR transduction, A2^pos^ CD8 T cells displayed substantially high expression of PD-1, TIM-3 and TIGIT co-inhibitory receptors and of CD25 and 4-1BB activation markers, again contrasting to the reduced CD28 expression, along the TCR affinity range (Fig. 3 c, Additional file 1: Figure S3A). These A2^pos^ CD8 T cells also co-expressed multiple inhibitory receptors (Fig. 3 d), but still showed enhanced relative proliferation capacity (Additional file 1: Figure S3B). Conversely, knock-out of A2 expression completely abolished this phenotypic activation state. These results show that A2 expression per se (in the absence of cognate antigen) is required to induce the sustained activation phenotype of tumor-redirected primary CD8 T cells upon affinity-increased TCR transduction.

### A CDR3-based TCR variant of improved binding avidity displays phenotypic activation and TCR/CD3 downmodulation

Since the above-described experiments were performed on TCR-engineered CD8 T cells, which mainly rely on amino-acid changes within the CDR2α/β loops, we generated two additional TCR variants containing mutations only in CDR3α/β (Additional file 1: Table S1). Redirected CD8 T cells with the α95:LYm TCR variant, currently used in clinical trials [2–4], depicted TCR-pMHC half-lives that were slightly inferior to those obtained with the DMβ TCR one (Fig. 3 e, Additional file 1: Table S1), as measured by the NTAmer-based dissociation assay [23]. Consequently, we designed the α95:LYm/A97L TCR variant, that combines dual CDR3α (α95:LYm) and single CDR3β (A97L) substitutions. This variant enabled reaching TCR-pMHC off-rate values similar to those found for the optimal TCR binding range (i.e. between DMβ and TMβ) (Fig. 3 e). Under baseline conditions, increased PD-1, TIM-3 and CD69 expression were observed for A2^pos^ primary CD8 T cells engineered with the α95:LYm/A97L TCR variant, with only a trend for the α95:LYm bearing cells (Fig. 3 f). This phenotypic activation was further associated to the downmodulation of CD28 (Fig. 3 f) and of TCR/CD3ε complex, while CD5 expression was enhanced (Fig. 3 g). Together, the CDR3-based TCR variant (α95:LYm/A97L) displayed comparable phenotypic activation and TCR/CD3 downmodulation as seen for TMβ, bearing dual and single point-mutations in CDR2β and CDR3β (A97L), respectively (Additional file 1: Table S1).

### A2 expression is linked to functional hyporesponsiveness through affinity-increased TCRs

**Fig. 4:**
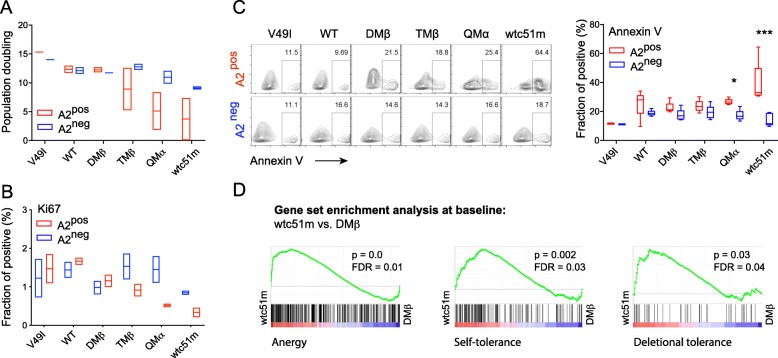
Proliferative capacity, basal apoptotic levels and tolerance-related transcriptional profiles in relation to HLA-A2. **a** Population doublings of A2^pos^ and A2^neg^ tumor-redirected primary CD8 T cells upon CRISPR/A2-Cas9 strategy were assessed by periodic cell counting of living cells during 10 days post-TCR sorting in the absence of cognate antigen. Data are representative of 2 independent experiments. **b, c** Quantification of the fraction of Ki67-positive (**b**) and Annexin-V-positive cells (**c**) in A2^pos^ (CRISPR/GFP) and A2^neg^ (CRISPR/A2) primary CD8 T cells under resting conditions. **c** Representative dot blots (left panel) and Annexin V quantifications (right panel) are depicted. Data are means ± SD of 4 to 5 independent experiments. * P ≤ 0.05 and *** P ≤ 0.001. **d** GSEA of available gene sets describing anergy [24], self-tolerance [25], and deletional tolerance [26] were found enriched in A2^pos^ wtc51m- versus DMβ-expressing primary CD8 T cells under steady-state culture conditions. Nominal *P* values and false discovery rates (FDR) are indicated for each gene set enrichment

We next investigated whether this step-wise activation to TCR downmodulation could further impact on the functional capacity of redirected A2^pos^ CD8 T cells of increased affinity TCRs. Following TCR transduction (> day 15), A2^pos^ (i.e. CRISPR/mock) and A2^neg^ (i.e. CRISPR/A2) primary CD8 T cells were FACS-sorted, non-specifically expanded by PHA/A2^neg^ feeder cells, before being evaluated for cell proliferation capacity and basal apoptotic levels (Fig. 3 b). All A2^neg^ redirected CD8 T cell variants displayed similar population doublings, contrasting to the impaired proliferative potential of A2^pos^ T cells along the TCR affinity gradient (Fig. 4 a). This correlated to reduced Ki67^pos^ (Fig. 4 b) and increased Annexin-V^pos^ (Fig. 4 c) cell fraction. Since PD-1 was rapidly upregulated upon increased-affinity TCR transduction and could account for this T cell hyporesponsive state, we incubated A2^pos^ redirected T cells with anti-PD-1 mAb (nivolumab) from day − 1 of TCR transduction throughout the whole experimental timeline as indicated in Fig. 3 a and b. Strikingly, PD-1 blockade did not prevent the early activation phenotype nor the subsequent reduced proliferative capacity of the high affinity A2^pos^ T cells (Additional file 1: Figure S4). We also performed a gene set enrichment analysis (GSEA) and found that gene sets associated to anergy, self-tolerance and deletional tolerance [24–26] were preferentially enriched in the high-affinity (i.e. wtc51m) TCR bearing A2^pos^ T cells compared to the wild-type or optimal-affinity (i.e. DMβ) TCR-expressing cells (Fig. 4 d, Additional file 1: Figure S5), under steady-state conditions (Additional file 1: Table S3).

**Fig. 5:**
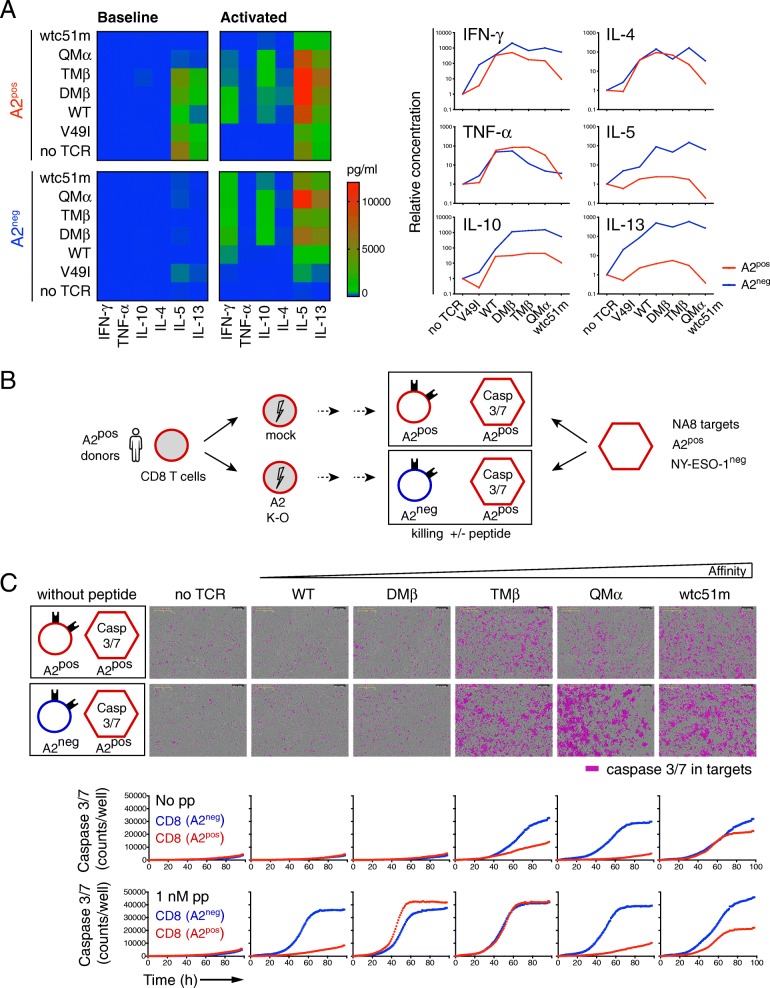
Basal cytokine production and killing capacity in relation to HLA-A2. **a** Multiplex cytokine production data was performed on A2^pos^ and A2^neg^ primary CD8 T cells without (baseline) or with low dose of 0.1 nM A2/NY-ESO-1_157-165_ multimers (activated) during 24-h of culture. Cytokine concentrations are shown as heat map (left panel) or as relative concentrations to no-TCR variants after specific stimulation (right panel). **b** Schematic representation of the real-time quantitative killing assay by IncuCyte. A2^pos^ (CRISPR/GFP) and A2^neg^ (CRISPR/A2) primary CD8 T cells of affinity-increased TCRs were co-cultured with NA8 (A2^pos^/NY-ESO-1^neg^) target tumor cells (E:T ratio; 1:10), without or with low dose of 1 nM NY-ESO-1_157-165_ peptide (pp) during 4 days. **c** Representative images at 96 h (upper panels) and quantification (lower panels) of caspase 3/7-dependent apoptosis induced by tumor-redirected A2^pos^ versus A2^neg^ CD8 T cells are shown. Data are representative of 2 independent experiments

Furthermore, we evaluated the impact of sustained TCR affinity-mediated cell activation on the ability of A2^pos^ versus A2^neg^ tumor-redirected primary CD8 T cells to produce various cytokine mediators by multiplex cytokine profiling. Data showed reduced Th1 and Th2 cytokine production in high-affinity A2^pos^ T cells compared to the A2^neg^ cells after low dose NY-ESO-1/multimer stimulation (Fig. 5 a). A similar trend was observed under resting conditions. We further found that A2^neg^ CD8 T cells of high affinity TCRs displayed increased baseline levels of granzyme B and perforin, as well as stronger killing or CD107a degranulation capacity in peptide-pulsed target assays than the corresponding A2^pos^ T cells (Additional file 1: Figure S6A-C). Despite these functional changes, the EC_50_ values defined as the peptide concentration producing half-maximal response, were for each defined TCR variant similar between A2^pos^ and A2^neg^ T cells, indicating that the sensitivity of TCR triggering for a given TCR affinity variant was fully preserved (Additional file 1: Figure S6D). Finally, we performed real-time quantitative killing analyses by incubating A2^pos^ or A2^neg^ redirected T cells together with NA8 melanoma cells (NY-ESO-1^neg^/A2^pos^) over a 4-day period using IncuCyte technology (Fig. 5 b). This approach also enabled us to address whether chronic TCR-A2 (self)-interactions could lead to qualitative functional adjustments over time. In the absence of cognate antigen, NA8/A2^pos^ target killing was observed for both A2^pos^ and A2^neg^ T cells with increased-affinity TCRs (i.e. TMβ, QMα and wtc51m) (Fig. 5 c). Nonetheless, A2^neg^ T cells always displayed better killing capacity over A2^pos^ T cells. The adjunction of low dose of NY-ESO-1 peptide allowed the functional stimulation of all TCR-redirected A2^neg^ T cells, irrespective of TCR affinity, whereas improved A2^pos^ T cell-mediated killing only occurred for TCR variants with affinities in the upper natural range (DMβ, TMβ). Together, these data indicate that A2^pos^ tumor-redirected primary CD8 T cells of affinity-increased TCRs are characterized by the upregulation of tolerance-like gene sets (Additional file 1: Table S3) combined to an overall functional impairment (i.e. proliferation, cytokine production and killing capacity) in comparison to A2^neg^ T cells. This functional hyporesponsiveness was retained following low doses of antigen-specific stimulation (Fig. 5), in line with our previous observations [9, 17].

### Short-term TCR-A2 (self)-interactions *in trans* lead to phenotypic and functional T cell activation

**Fig. 6:**
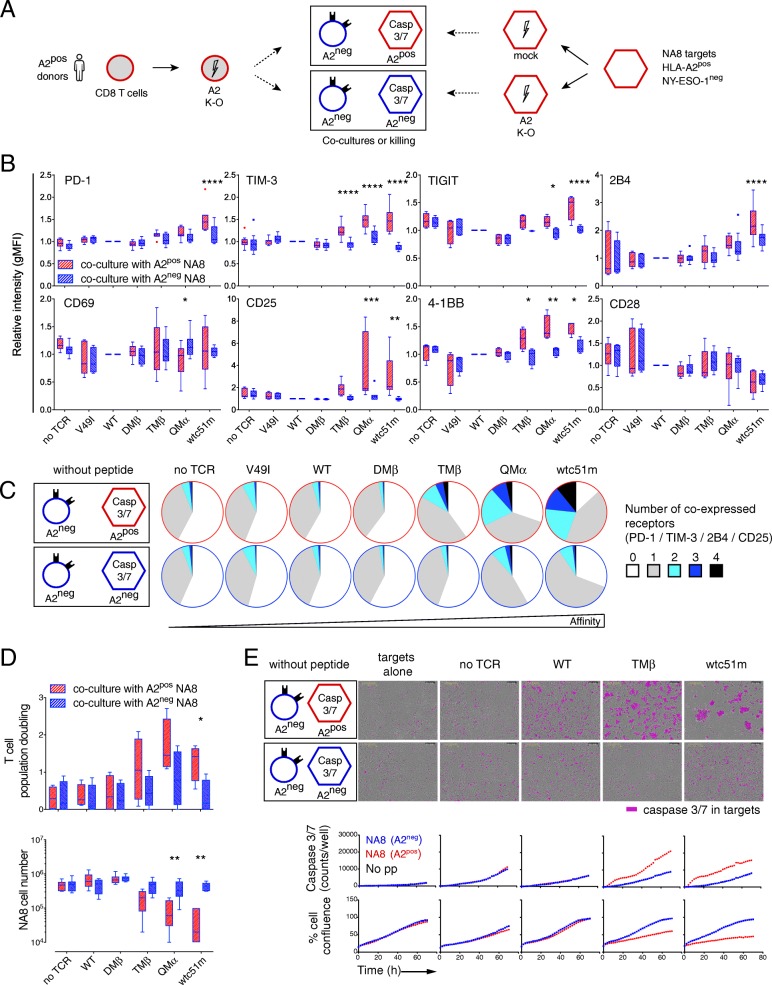
Activation phenotype and basal killing capacity of tumor-redirected A2^neg^ CD8 T cells in short-term co-cultures with NA8 target cells. **a** Schematic representation of the experimental design; A2^neg^ (CRISPR/A2) primary CD8 T cells of increased-affinity TCRs were co-cultured with A2^pos^ or A2^neg^ (CRISPR/A2) NA8 tumor cells for 3 days in the absence of cognate antigen. **b** Expression levels of co-activating/inhibitory receptors on A2^neg^ CD8 T cells after 3 days of co-culture with either A2^pos^ or A2^neg^ NA8 cells. **c** Co-expression of 0 to 4 co-inhibitory (PD-1, TIM-3 and 2B4) and co-activating (CD25) receptors. **d** Quantification of T cell population doublings (upper panel) and of NA8 cell numbers (lower panel) after 3 days of co-culture with A2^pos^ or A2^neg^ NA8 cells. **e** Representative images at 70 h (upper panel) and quantification (lower panel) of caspase 3/7-dependent apoptosis induced by tumor-redirected A2^neg^ CD8 T cells co-cultivated during 3 days with A2^pos^ or A2^neg^ NA8 cells are depicted, using IncuCyte technology. Data are expressed as means ± SD and are representative of 4 to 8 (**b** and **c**), 4 to 6 (**d**) and 2 (**e**) independent experiments. * P ≤ 0.05, ** P ≤ 0.01, *** P ≤ 0.001 and **** *P* ≤ 0.0001

Given the importance of A2 expression on the step-wise activation-to-hyporesponsive state of A2^pos^ CD8 T cells, we hypothesized that A2-(self) molecules per se may directly trigger chronic interactions with affinity-increased TCRs. To address this question, we initially performed short-term (72 h) co-cultures of A2^neg^ primary CD8 T cells with either A2^pos^ (CRISPR/mock) or A2^neg^ (CRISPR/A2) NA8 target cells in the absence of cognate antigen (Fig. 6 a). Multiple co-activating (CD25, 4-1BB) and co-inhibitory (PD-1, TIM-3, TIGIT and 2B4) receptors were found up-regulated and co-expressed when T cells of affinity-increased TCRs were cultivated in presence of A2^pos^ NA8 cells (Fig. 6 b and c). Cocultures between A2^neg^ J76 CD8αβ T cells and A2^pos^ NA8 cells led to similar PD-1 and CD69 upregulation (Additional file 1: Figure S7). Moreover, this activation state correlated to enhanced proliferation and killing capacity of the T cells when co-cultivated with A2^pos^ but not A2^neg^ NA8 cells (Fig. 6 d and e). Along the same lines, mixing A2^pos^ (CRISPR/mock) and A2^neg^ (CRISPR/A2) primary CD8 T cells together resulted in the progressive disappearance over time of A2^pos^ T cells, inversely associated to the enrichment of A2^neg^ NY-ESO-1-specific T cells, in high affinity TCR variants (Additional file 1: Figure S8). These data demonstrate that short-term TCR-A2 (self)-interactions *in trans* can induce the phenotypic and functional activation of A2^neg^ CD8 redirected T cells of affinity-increased TCRs (i.e. from TMβ onwards), in the absence of cognate antigen.

### Long-term TCR-A2 (self)-interactions *in trans* drive a functional hyporesponsive state

**Fig. 7:**
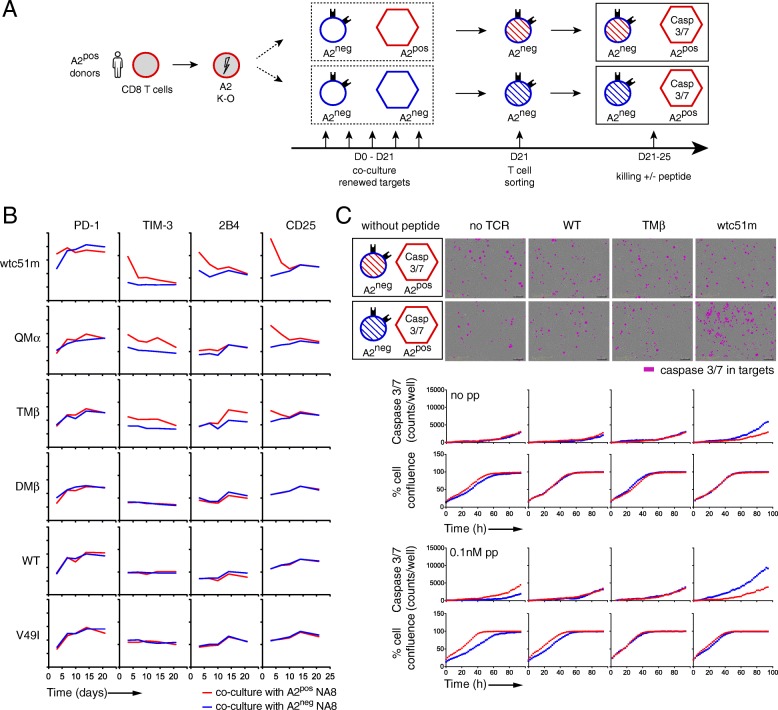
Activation phenotype and basal killing capacity of tumor-redirected A2^neg^ CD8 T cells in long-term co-cultures with NA8 tumor cells. **a** Schematic representation of the experimental design; A2^neg^ (CRISPR/A2) primary CD8 T cells were co-cultured with either A2^pos^ or A2^neg^ NA8 tumor cells for 20 days in the absence of cognate antigen before being sorted and tested for their killing capacity. **b** Kinetics of the expression levels of co-activating/inhibitory receptors on A2^neg^ CD8 T cells during long-term co-cocultures with A2^pos^ or A2^neg^ NA8 cells. **c** Representative images at 94 h (upper panel) and quantification (lower panel) of caspase 3/7-dependent apoptosis of NA8^pos^ target cells induced by A2^neg^ CD8 T cells previously co-cultured during 20 days with either A2^pos^ or A2^neg^ NA8 cells are depicted, using IncuCyte

To investigate whether chronic TCR-A2 (self)-interactions could lead to the previously observed hyporesponsive state (Figs. 4 and 5), we performed extended co-cultures between A2^neg^ primary CD8 T cells and either A2^pos^ or A2^neg^ NA8 cells, by passing the T cells over fresh layers of tumor cells every 3–4 days (Fig. 7 a). All co-cultures were again carried out in the absence of antigen-specific stimulation. Kinetic analyses showed that following the rapid and initial upregulation of co-activating and co-inhibitory receptors on CD8 T cells redirected with high affinity TCRs, there was a progressive decline in their expression thereafter (Fig. 7 b). This effect was only found in co-cultures with A2^pos^ NA8 cells. Cell proliferation was no longer noticed for increased-affinity T cells after day 12 of co-culture with A2^pos^ NA8 cells (data not shown). We also examined the killing capacity of these long-term cultured A2^neg^ CD8 T cells against A2^pos^ NA8 target cells using the IncuCyte design (Fig. 7 c). The high affinity T cell variant (wtc51m), cocultured during extended periods of time with A2^neg^ NA8 cells, retained superior killing capacity over WT or TMβ T cell variants. This was not the case when the same wtc51m A2^neg^ T cells were previously co-cultivated with A2^pos^ NA8 cells over time, as this co-culture combination led to impaired T cell killing capacity (Fig. 7 c). Collectively, the long-term exposure to A2 expression *in trans* recapitulates at the killing level, the observed hyporesponsive state found in A2^pos^ T cells upon high affinity TCR transduction. These results further indicate that chronic TCR-A2 (self)-interactions can trigger sustained T cell activation, driving to the functional hyporesponsiveness in CD8 T cells engineered with affinity-increased TCRs.

## Discussion

TCR-ligand interactions influence many aspects of T cell biology. Stronger interactions usually confer superior T cell activation and responsiveness than weaker ones [27–30]. However, recent advances describe that negative feedback mechanisms may limit effector function according to TCR affinity/avidity [9, 11–13]. Here, we addressed the question whether increasing the TCR affinity can directly trigger the chronic TCR recognition of A2-(self) molecules (i.e. TCR-MHC (self)-interactions) and modulate the overall functional potency of tumor-redirected CD8 T cells. Using two complementary tumor-redirected CD8 T cell models, we first showed that the de novo expression of A2 molecules in TCR-engineered Jurkat J76 CD8αβ T cells led to TCR/CD3 downregulation and impaired TCR signaling in a TCR affinity-dependent manner (Fig. 1). This was inversely correlated to enhanced expression of the negative TCR tuning molecules, CD5 and c-CBL (Fig. 1). We further found that strong T cell activation always preceded global T cell hyporesponsiveness in tumor-redirected primary CD8 T cells of increasing affinity TCRs (Fig. 2-5). This was again dependent on the recognition of A2 molecules and was already observed for TCR variants engineered with affinities lying at the upper limit of the physiological range. Finally, the stepwise activation-to-hypofunctional state could be recapitulated *in trans* in A2^neg^ primary CD8 T cells of high affinity TCRs when co-cultured with A2^pos^-presenting NA8 cells (Figs. 6 and 7). Together, our data indicate that chronic interactions between affinity-increased TCRs and self-A2 molecules can directly tune the functional potential of CD8 T cells, even in the absence of antigen-specific stimulation. This TCR affinity-mediated hyporesponsive state is novel and has implications for the design of affinity-improved TCRs for immunotherapy.

Our findings are in agreements with recent studies reporting that chronic activation arising independently of antigenic stimulation can drive a hyporesponsive functional state in primary T cells [31, 32]. Namely, enhancing basal TCR signaling with a Zap-70 gain-of-function mutation was associated with marked increase in PD-1 expression and T cell unresponsiveness, a state sharing features to T cell anergy [31]. Moreover, tonic CD3ζ phosphorylation triggered through the clustering of chimeric antigen receptors (CARs) predisposes the CAR-T cells to early exhaustion and limits their in vivo efficacy against tumors [32]. Here, we describe that TCR-A2 (self)-interactions occurring with affinity-increased TCRs provided sustained activation stimuli to transduced CD8 T cells, resulting in subsequent impaired functional performance. These high affinity T cells also shared several features commonly seen in other hyporesponsive T cell states such as exhaustion [33]. For instance, they co-expressed several inhibitory receptors, before displaying gene sets related to self-tolerance and showing reduced ability to proliferate and to produce cytokines (Figs. 3 4 and 5). These observations offer new evidence that chronic T cell activation engaged negative feedback regulations by which inhibitory receptors and TCR/CD3 downmodulation likely restrain TCR signaling and function, to provide potential protective mechanisms against TCR-MHC self-reactivity.

One possible explanation for the chronic interactions occurring between affinity-increased TCRs and A2 (self)-molecules might stem from the TCR affinity-optimization process used to generate our NY-ESO-1-specific TCR panel [18], including the nanomolar affinity TCR wtc51m variant, designed by phage-display screening [34]. Gain in TCR affinity above the physiological range (K_D_ ≤ 1 μM) was mostly related to amino-acid changes involving mutations in CDR2α/β combined to single point-mutations within CDR3β [9, 17]. As in other TCR-pMHC systems, in our TCR model the CDR1 and CDR2 are likely to primarily interact with MHC moieties, whereas the CDR3 largely interacts with the peptide [35, 36]. Hence, the observations described here are consistent with the concept that in redirected T cells of increased-affinity TCRs, continuous TCR-MHC (self)-interactions leading to the upregulation of regulatory feedback mechanisms may notably occur via the amino acid modifications generated at the CDR2 loops. An alternative hypothesis is based on the following model proposing that T cells may only naturally function in a well-defined affinity range to ensure optimal responses while preventing T cell-mediated overreactive ones [37]. Indeed, numerous studies using human or mouse models and relying on affinity-optimized TCR variants or altered-peptide ligands indicate that maximal T cell activation and functional potency occurs at intermediate TCR-pMHC binding affinities or half-lives (reviewed in [7]). Our results are also in line with this point of view. In the present study, we describe that upstream regulatory mechanisms such as TCR/CD3 downregulation and co-expression of multiple inhibitory receptors may restrict T cell functional potency according to the TCR affinity for self-MHC molecules. These TCR affinity-associated regulations were even observed for the TMβ variant possessing a TCR affinity lying at the higher end of the physiological range. Interestingly, similar findings were obtained when designing a TCR variant (i.e. α95:LYm/A97L) containing point-mutations exclusively within the CDR3α/β loops (Fig. 3). These observations are suggestive of the presence of TCR affinity-related activation thresholds. At present, additional studies are still needed to fully appreciate the respective consequences of CDR3α/β-based mutations compared to those involving the CDR2α/β ones, on the overall TCR interactions to HLA-A2, the functional efficacy and the potential off-target recognition of these tumor-redirected CD8 T cells.

The impact of chronic TCR-A2 (self)-interactions on tumor-redirected A2^pos^ CD8 T cells upon TCR transduction of increased affinities led to the co-expression of multiple inhibitory receptors such as PD-1, TIM-3, TIGIT and 2B4, that preceded T cell hyporesponsiveness (Fig. 3). This is reminiscent of the recently developed concept that many inhibitory receptors including PD-1 represent markers of T cell activation [38]. Notably, PD-1 levels have been related to the strength of TCR signaling and thus to the functional avidity of tumor-specific T cells to compensate for T cell activation [39]. Another finding was that early PD-1 inhibition by nivolumab did not reverse the stepwise activation to hypofunctional state of our TCR affinity-optimized A2^pos^ CD8 T cells (Additional file 1: Figure S4). These observations suggest that the hyporesponsive state of increased affinity CD8 T cells may involve the combination of several distinct negative regulatory pathways. In support to this, we found that basal TCR/CD3ε downmodulation inversely correlated to increased expression of the tuning molecules CD5 and c-CBL [20], and resulted into additional impaired TCR signaling (Figs. 1 and 2). Intriguingly, early PD-1 blockade did not recapitulate the results obtained upon PD-L1 blockade in long-term cultured hyporesponsive A2^pos^ T cells of highest affinity TCRs and showing functional recovering [9]. One possible explanation is that as PD-1 expression is finely regulated by genetic and epigenetic dynamic mechanisms [40], the stage at which CD8 T cells can get reinvigorated upon PD-1 blockade may matter. In that regard, late cultures of CD8 T cells may be more sensitive to the inhibition of the PD-1/PD-L1 axis than early expanded T cells upon TCR transduction. Alternatively, blocking PD-1 directly (as shown here) may not necessarily lead to the same biological effects than PD-L1 inhibition [9], since PD-1 and PD-L1 may be differently expressed on given T cell subpopulations and their expression may further differ over time after activation [41].

One major safety concern when using affinity-enhanced TCRs for adoptive T cell therapy, is that candidate TCRs may target normal tissues as a consequence of off-target recognition [1]. Our study further emphasizes the possible impact of TCR-MHC (self)-interactions in relation to membrane receptor expression, cell activation, signaling and function of redirected CD8 T cells. The development of various safeguard strategies as for example the use of more complex cell cultures [6], is therefore becoming mandatory in preclinical studies to provide a better evaluation of these potential adverse risks. In that regard, Inderberg and colleagues recently tested the effect of overexpressing the negative regulator c-SRC kinase in redirected T cells and showed that while the T cells retained target recognition and binding, they were incapable of executing their effector functions [42]. They propose to use these “dummy” T cells for in vivo safety validation of new therapeutic TCRs prior their clinical use [42]. Here, we show that the J76 cell line, devoid of endogenous TCRαβ chains and expressing CD8αβ coreceptor represents another useful model to appraise the impact of increased-affinity TCRs and MHC expression on TCR/CD3 complex activation and signaling capacity (Fig. 1). TCR-redirected primary CD8 T cells expressing A2 (CRISPR/GFP) or not (CRISPR/A2) are also highly indicative of how given affinity-increased TCRs do recognize and kill target cells in the absence of cognate antigen (Fig. 5). Notably, the use of primary CD8 T cell lacking A2 expression provided a complementary evaluation of the potential TCR-MHC (self)-interactions occurring in relation to TCR affinity. The IncuCyte technology further offers a strong biological and relevant culture system as it allows assessing the long-term functional impact of affinity-engineered TCRs on target cells at the qualitative level. Investigations based on animal models still remain necessary, as these cannot be fully replaced by in vitro studies, especially in validating the in vivo capacity of engineered T cells. Yet, mouse models may not always be suitable for predicting off-target toxicities, as demonstrated in the preclinical investigations performed on the affinity-enhanced MAGE-A3/HLA-A1 TCR that caused fatal toxicity against cardiac tissues [6].

## Conclusions

This report, together with others [43, 44], underlines the importance of antigen-specific TCR recognition for self-MHC in calibrating subsequent T cell specificity or selection. TCR-MHC (self)-interactions may contribute as a sensor leading to peripheral tolerance and preventing excessive auto-reactivity. Specifically, our work highlights the consequence of TCR-A2 (self)-interactions related to TCR affinity on the activation, signaling and functional potency of tumor-redirected CD8 T cells. It also shows that the use of TCR-redirected J76 CD8αβ T cells combined to primary CRISPR/A2 CD8 T cells directly contributed to the assessment of these self-interactions in the absence of cognate peptide, which could potentially affect the anti-tumor T cell responses in vivo. Importantly, all of these aspects must be carefully weighted, especially in the frame of recent genetic editing strategies promoting the selective elimination of HLA-class I expression to generate universal T cells from allogeneic donors [45]. Together, this study further underlines the need to wisely assess TCR affinity-increased candidates for ensuring optimal and harmless TCR design for adoptive T cell-based therapies.

## Supplementary information

**Additional file 1.** **Table S1.** Characteristics of affinity-increased HLA-A2/NY-ESO-1-specific TCRs. **Table S2.** List of antibodies 93 used in the study. **Table S3.** List of gene 95 sets used for GSEA. **Figure S1.** Characterization and baseline expression of TCR/CD3 complex in de novo HLA-A2-expressing Jurkat J76 CD8αβ T cells. **Figure S2.** Phosphorylation levels of CD3ζ and ERK upon stimulation and basal expression levels of surface receptors in A2pos and A2neg J76 CD8αβ T cells. **Figure S3.** Basal expression levels of co-activating/inhibitory receptors on A2pos and A2neg primary CD8 T cells upon affinity-increased TCR transduction. **Figure S4.** Surface staining and population doublings of primary 130 A2pos CD8 T cells with or without PD-1 blocking antibody. **Figure S5.** Gene Set Enrichment Analysis (GSEA) of high-affinity versus optimal TCR variants. **Figure S6.** CD107a degranulation and killing capacity of A2pos and A2neg primary CD8 T cells expressing affinity-increased TCRs. **Figure S7.** Expression levels of PD-1 and CD69 in A2neg J76 CD8αβ cells during 14 days of co-culture with NA8 tumor cells. **Figure S8.** Dynamics of A2pos versus A2neg redirected primary CD8 T cell sub-populations in co-cultures following TCR transduction.

## Data Availability

The datasets used and/or analyzed during the current study are available from the corresponding authors.

## Abbreviations

A2: HLA-A*0201
MAPK: Mitogen-activated protein kinases
PD-1: programmed cell death-1
PD-L1: PD-1 ligand 1
pMHC: peptide-Major Histocompatibility Complex
PTPN6/SHP-1: Protein tyrosine phosphatase non receptor type 6
TCR: T cell receptor
