# Supplementary material for: Chronic TCR-MHC (self)-interactions limit the functional potential of TCR affinity-increased CD8 T lymphocytes
Source: J Immunother Cancer. 2019 Nov 5;7:284. doi: 10.1186/s40425-019-0773-z (PMC6833194; doi:10.1186/s40425-019-0773-z)
Supplement: Supplementary file 1 — Additional file 1. Table S1. Characteristics of affinity-increased HLA-A2/NY-ESO-1-specific TCRs. Table S2. List of antibodies 93 used in the study. Table S3. List of gene 95 sets used for GSEA. Figure S1. Characterization and baseline expression of TCR/CD3 complex in de novo HLA-A2-expressing Jurkat J76 CD8αβ T cells. Figure S2. Phosphorylation levels of CD3ζ and ERK upon stimulation and basal expression levels of surface receptors in A2pos and A2neg J76 CD8αβ T cells. Figure S3. Basal expression levels of co-activating/inhibitory receptors on A2pos and A2neg primary CD8 T cells upon affinity-increased TCR transduction. Figure S4. Surface staining and population doublings of primary 130 A2pos CD8 T cells with or without PD-1 blocking antibody. Figure S5. Gene Set Enrichment Analysis (GSEA) of high-affinity versus optimal TCR variants. Figure S6. CD107a degranulation and killing capacity of A2pos and A2neg primary CD8 T cells expressing affinity-increased TCRs. Figure S7. Expression levels of PD-1 and CD69 in A2neg J76 CD8αβ cells during 14 days of co-culture with NA8 tumor cells. Figure S8. Dynamics of A2pos versus A2neg redirected primary CD8 T cell sub-populations in co-cultures following TCR transduction. [file 40425_2019_773_MOESM1_ESM.pdf]

SUPPLEMENTAL DATA

SUPPLEMENTAL MATERIAL AND METHODS

Culture of cell lines and primary CD8 T lymphocytes

HLA-A2^{neg}/TCRαβ^{ko}/CD8αβ^{pos} Jurkat J76 T cells (defined thereafter as HLA-A2^{neg} J76 CD8αβ cells) were generated following transduction of CD8α- and CD8β-encoding plasmids into the TCR α and β knock-out HLA-A2^{neg} J76 T cell subline using retroviral vectors (kindly provided by Drs. I. Edes and W. Uckert; Max-Delbrück-Center, Berlin, Germany, *unpublished data*). HLA-A2^{neg}/J76 CD8αβ cells, HLA-A2^{pos}/TAP-deficient T2 cells (ATCC CRL-1992), HLA-A2^{pos}/NY-ESO-1^{neg} NA8 cells (CVCL-S599) and HLA-A2^{pos}/NY-ESO-1^{pos} Me275 cells (CVCL_S597) were cultured at 37°C and 5% CO_2 in RPMI 1640 medium supplemented with 10% fetal calf serum (FCS), 100 U/mL penicillin and 100 µg/mL streptomycin (Gibco). Primary CD8 T lymphocytes were positively enriched from peripheral blood mononuclear cells (PBMC) obtained from healthy donors using anti-CD8-coated magnetic microbeads (Miltenyi Biotec) and cultured at 37°C and 5% CO2 in RPMI supplemented with 8% human serum, 100 U/mL penicillin, 100 µg/mL streptomycin, 2 mM L-glutamine, 0.1 mg/mL kanamycin, 1 mM sodium pyruvate, 1X non-essential amino acids, 50 µM β-mercaptoethanol (Gibco) and 150 U/mL recombinant human IL-2 (gift from GlaxoSmithKline). HLA-A*0201 (A2 thereafter) status of the cells was determined by flow cytometry performed on PBMCs before CD8 isolation. A2^{pos} and A2^{neg} CD8 T lymphocytes were stimulated with CD3/CD28 beads (ThermoFisher) at a ratio of 1:1 for transduction and expansion, and subsequently expanded every 14-21 days by re-stimulation with 1 µM phytohemagglutinin (PHA; Oxoid) and 30 Gy-irradiated allogeneic A2^{neg} PBMCs as feeders.

Generation of CRISPR-A2 primary CD8 T cells and CRISPR-A2 NA8 tumor cells

The kinetic experiments (see Fig. 2, main manuscript) were performed on primary CD8 T cells from A2^{pos} or A2^{neg} healthy donors, for which donor-specific variations could be observed in the *ex vivo* expression of surface receptors (data not shown). Therefore, using the CRISPR/Cas9 technology, we generated A2^{pos} (i.e. CRISPR/mock) and A2^{neg} (i.e. CRISPR/A2) primary CD8 T cells sharing the same cellular background. CRISPR-A2 primary CD8 T cells and NA8 cells were produced according to the protocol by Ran et al. (1). Briefly, the 20 nucleotide-single guide (sgRNA) sequence targeting HLA-A*0201 was designed using http://CRISPR.mit.edu website and selected for high quality score (>80) to minimize off-targets. The sequence of the sgRNA is as following: GAGGGTCCGGAGTATTGGGA. An extra 5' G nucleotide was added

to the sgRNA sequence to improve U6 promoter efficiency. The oligo pairs encoding the sgRNA were annealed and ligated into a BsmBI-digested lenti-CRISPR plasmid v2 bearing Cas9 and sgRNA scaffold (Addgen plasmid #52961) to generate lenti-CRISPR-A2 plasmid. This plasmid was transfected into HEK 293T/17 cells (ATCC CRL-11268) using a standard calcium phosphate protocol for the production of lentiviral particles. The supernatant was concentrated by ultracentrifugation at 24000 g for 2h and subsequently used to infect freshly isolated A2^{pos} CD8 T lymphocytes after 24h stimulation with CD3/CD28 beads (1st expansion) or NA8 cells to generate CRISPR-A2 (A2^{neg})-CD8 T cells or CRISPR-A2 (A2^{neg})-NA8 cells, respectively. Lenti-CRISPR-EGFP sgRNA 6 (Addgen plasmid #51765) was used as a mock control. Transduced cells (A2^{neg}) were sorted to purity with PE-labeled HLA-A2 antibody by flow cytometry (FACSAriaII, BD Biosciences).

Generation of TCR-engineered primary CD8 T cells and J76 CD8αβ T cells

The full-length codon-optimized TCR AV23.1 and TCR BV13.1 chain sequences of a dominant NY-ESO-1$_{157\text{-}165}$-specific T cell clone of patient LAU155 (BC1) were cloned in the pRRL lentiviral vectors, as IRES or T2A constructs. Structure-based amino acid substitutions within CDR2αβ and/or CDR3αβ loops were introduced into the wild-type (WT) TCR sequence using the QuickChange mutagenesis kit (Stratagene) and all mutations were confirmed by DNA sequencing (2, 3). Concentrated supernatant of lentiviral-transfected 293T cells was used to infect (i) A2^{pos} and A2^{neg} primary CD8 T cells (1st expansion), (ii) CRISPR-A2 and CRISPR-EGFP primary CD8 T cells (2nd expansion) or (iii) A2^{pos} and A2^{neg} J76 CD8αβ T cells. Primary CD8 T cells were infected overnight in plate coated with 10 μg/cm^2 retronectin (Takara), while J76 CD8αβ cells were infected for 30 min at 37°C. The transduction efficiency was 5-30% for freshly isolated CD8 T cells, 1% for 2nd round of stimulated CRISPR-CD8 T cells, and over 96% for J76 CD8αβ cells. Integrated TCR lentiviral copy number was quantified by qPCR and was similar across TCR variants (1-2 copies/genome in primary sorted CD8 T cells and 9-26 copies/genome in J76 CD8αβ T cells) (data not shown; Supp. Table 1).

Granzyme B and perforin staining by flow cytometry

For intracellular staining, 3x10^5 TCR-transduced A2^{pos} and A2^{neg} primary CD8 T cells from healthy individuals were fixed in PBS 1% formaldehyde, 2% glucose, and 5 mM NaN_3 for 20 min at room temperature, before being stained with corresponding antibodies (granzyme B and perforin) in PBS 0.2% BSA, 5 mM EDTA, 0.2% NaN_3, and 0.1% saponin. All experiments were performed under unstimulated, resting culture conditions. Samples were acquired with a

Gallios (Beckman Coulter) flow cytometer and data were analyzed by FlowJo software (Tree star, v10.0.8).

CD107a degranulation assay

A2^{pos}/TAP-deficient T2 cells were pulsed with 0.1 μM native NY-ESO-1$_{157-165}$ peptide for 1h at 37°C, washed and incubated with TCR-transduced A2^{pos} or A2^{neg} primary CD8 T cells at an effector-to-target (E:T) of 2:1 for 4h in the presence of anti-CD107a/LAMP1 and 10 μg/mL brefeldin A. Samples were acquired with a Gallios (Beckman Coulter) flow cytometer and data were analyzed by Flowjo software (Tree star).

Chromium release assay

The antigen recognition capacity and *in vitro* lytic activity of primary A2^{pos} and A2^{neg} CD8 T lymphocytes engineered with TCRs of incremental affinities were assessed using chromium release assay as previously described (4). Briefly, HLA-A2^{pos}/TAP-deficient T2 cells or HLA-A2^{pos}/NY-ESO-1^{pos} Me275 cells were labeled with ^{51}Cr. In peptide titration assay, T2 cells were pulsed with serial dilution of native NY-ESO-1$_{157-165}$ peptide and incubated for 4 h with A2^{pos} or A2^{neg} CD8 effector T cells at E:T ratio of 10:1. In tumor killing assay, Me275 cells were pulsed or not with 1 μM of NY-ESO-1$_{157-165}$ native peptide and incubated for 4 h with A2^{pos} or A2^{neg} CD8 effector T cells at indicated E:T ratios. Percentage of specific lysis was calculated as 100x (experimental-spontaneous release)/(total-spontaneous release). Dose-response curve analysis and EC50 values were obtained using Prism software (GraphPad, v.7.03).

Functional PD1 blockade with nivolumab

One day prior to TCR transduction, primary A2^{pos} (directly isolated from a healthy donor) or A2^{pos} (isolated following CRISPR/GFP transduction) CD8 T cells were cultured without (control) or with 20 μg/mL PD-1 blocking antibody (nivolumab; a gift from the Department of Oncology, University Hospital Lausanne). The culture medium containing 20 μg/mL nivolumab was constantly renewed every 3-4 days.

Supplemental Table 1. Characteristics of affinity-increased HLA-A2/NY-ESO-1-specific TCRs

TCR name	NY-ESO-1-specific TCR (AV23.1/BV13.1) [a]				TCR-pMHC affinity&kinetics		quantification of TCR LV copy numbers # [e]			
	CDR2α	CDR3α	CDR2β	CDR3β	K_D (μM) [a]	$t_{1/2}$ (s) [d]	CD8αβ J76 cells [f]		primary CD8 T cells [f]	
	50 51 52 53 54 55	93 94 95 96 97 98	49 50 51 52 53 54	95 96 97 98 99 100			HLA-A2[pos]	HLA-A2[neg]	HLA-A2[pos]	HLA-A2[neg]
V49I	IQSSQR	RPQTGG	**I**GAGIT	VGAAGE	n.a.	10.7	19	26	2.3	2.2
WT [b]	IQSSQR	RPQTGG	VGAGIT	VGAAGE	21.4	53.0	39	18	1.4	1.9
A97L	IQSSQR	RPQTGG	VGAGIT	VG**L**AGE	2.69	102.2	n.d.	n.d.	n.d.	n.d.
DMβ	IQSSQR	RPQTGG	V**AE**GIT	VGAAGE	1.91	219.7	20	20	1.8	1.1
TMβ	IQSSQR	RPQTGG	V**AE**GIT	VG**L**AGE	0.91	453.1	18	9	1.2	1.9
QMα	IQS**W**QR	RPQTGG	V**AE**GIT	VG**L**AGE	0.14	479.7	16	12	1.4	1.3
wtc51m [c]	IQSSQR	RPQTGG	V**AIQT**T	VGAAGE	0.015	3043.0	20	14	1.0	1.2
α95:LYm [c]	IQSSQR	RP**LY**GG	VGAGIT	VGAAGE	n.a.	172.4	n.d.	n.d.	n.d.	n.d.
α95:LYm/A97L	IQSSQR	RP**LY**GG	VGAGIT	VG**L**AGE	n.a.	260.2	n.d.	n.d.	n.d.	n.d.

[a] Point-mutations (in red) and molecular affinity values (by SPR) of affinity-increased TCR variants as described in (3).

[b] The wild-type BC1 TCR was isolated from melanoma patient LAU155 (5) and differs from the 1G4 TCR (6) by only 4 aa residues (underlined).

[c] NY-ESO-1-specific variants containing the wtc51 (7) or α95:LY (8) mutations within the BC1 TCR background.

[d] Monomeric TCR-pMHC off-rates ($t_{1/2}$) measured by the cell surface dissociation assay at 4°C using two-color reversible NTAmers (9).

[e] Lentiviral genomic copy number quantified by qPCR of *gag* and normalized to *albumin.*

[f] Generation of TCR-transduced HLA-A2[pos] or HLA-A2[neg] CD8αβ J76 and primary CD8 T cells as described in the Material & Methods section.

Supplemental Table 2. List of antibodies used in the study

Targets	Color	Manufacturer	Reference
panTCRαβ	PC5	Beckman Coulter	A39500
panTCRαβ	PE	Beckman Coulter	B49177
Vbeta13.1	PE	Beckman Coulter	IM2292
CD3ε	PC5.5	BioLegend	300430
CD3ε	BrV421	BioLegend	300434
PD-1	APC	BioLegend	329908
PD-1	BrV421	BioLegend	329920
TIM-3	PC7	eBioscience	25-3109-42
TIGIT	APC	eBioscience	17-9500-42
2B4	PC5.5	BioLegend	329514
CD69	FITC	BD Biosciences	347823
CD25	BrV421	BioLegend	302629
4-1BB	PC7	BioLegend	309818
CD28	BrV421	BioLegend	302930
CD8β	PE	Beckman Coulter	IM2217U
CD8β	FITC	Beckman Coulter	B42025
CD5	BV421	BioLegend	300626
CD107a	FITC	BD Bioscience	555800
Annexin V	Cy5	BD Bioscience	559933
Ki67	FITC	BD Bioscience	556026
Granzyme B – intracellular	FITC	BioLegend	515403
Perforin – intracellular	APC	BioLegend	308112
c-CBL – intracellular	--	Abcam	Ab32027
Secondary anti-Rabbit IgG (for c-CBL)	FITC	BD Biosciences	554020
pCD3ζ (Y142) – phospho-flow	Alexa Fluor 647	BD Biosciences	558489
pERK71/2 (T202/Y204) - phospho-flow	Alexa Fluor 647	Cell Signaling	4375S

94 **Supplemental Table 3. List of gene sets used for GSEA**

SELF TOLERANT MOUSE CD8 (10)	https://www.ncbi.nlm.nih.gov/pubmed/22267581 (CLUSTER 9 and 13)	CD81, CLSPN, CDC20, KIF15, TCF19, LOC237877, LAG3, TNFRSF9, AURKA, BCAT1, TFRC, INCENP, MCM10, HMGN3, SNN, ESPL1, HIST1H3E, CHTF18, HIST1H2BN, TNFRSF9, KNTC1, GTSE1, SGOL1, ECM1, 3000004C01RIK, HIST1H3A, TFRC, KIF2C, CDCA2, FANCD2, 2410015N17RIK, CDC6, CHAF1A, CHST2, SPAG5, HIST1H2BM, CLDN10, IGF2BP3, NHEDC2, APITD1, CCDC99, LIG1, STMN1, ZRANB3, CDC45L, MARCKSL1, RGS16, PTPLA, CHST3, CENPM, HMGN3, CDC20, PTPRS, NCAPD2, NRGN, CENPM, HIST1H3C, SOX5, HIST1H3D, CKB, NRN1, HIST1H2BH, RCC1, BUB1B, HIST1H2BJ, HIST1H2AB, TPI1, MCM5, PTPRS, NMRAL1, RAD51, CDCA7, SPC25, NDRG1, KIF4, RILPL2, PIF1, CENPM, CENPA, RAD54L, HIST1H3H, 2610510J17RIK, HIST1H2BK, HIST1H2BF, E130016E03RIK, CDC7, ITM2A, FIBCD1, XCL1, SH3BP2, TOP2A, HIST1H2AF, PPIC, HIST1H2AN, CDC2A, BIRC5, HIST1H2AG, HIST2H2AB, PPIC, LITAF, E2F2, PRC1, CDCA3, H2AFX, BIRC5, HIST1H2AH, NUSAP1, PPA1, TYMS, LOC100047934, CCNB1, PSAT1, BIRC5, PBK, HIST1H2AK, MCM6, HIST1H2AD, TYMS-PS, TPI1, UHRF1, KLRB1C, ADK, RRM1, HIST1H2AO, E2F2, TNFRSF21, 2810417H13RIK, CCNB1, SCAMP1, CHN2, LMNB1, ANLN, RRM2, PKM2, KIF22, RRM2, PLK1, MAD2L1, LOC100045304, NCAPH, E2F1, HIST2H2AC, MCM6, TK1, FEN1, CLSPN, FIGNL1, D17H6S56E-5, NDRL, CDCA3, MCM2, KIF11, CDCA5, CDC20, CHAF1B, CAPG, 3000004C01RIK, MCM10, ENDOD1, CENPH, GALK1, SWAP70, LOC100047651, GFOD1
DELETIONAL TOLERANCE MOUSE CD8 (11)	https://www.ncbi.nlm.nih.gov/pubmed/19204323 (TABLE 1)	TNFSF11, RGS16, NRN1, IKZF2, TBC1D4, GPM6B, ENDOD1, FCRL1, LRIG1, LRIG1, MARCKS, NR4A3, LTA, MARCKS, SYNJ2, NR4A1, HSPA4L, PTPRS, EGR2, PSCD3, YPEL2, EGR2, SKIL, NAB2, SOAT1, GALM, TOX, 4930539E08RIK, REEP3, MDH1, 4921525O09RIK, EPHX1, SSH1, CREM, PACSIN1, LPCAT1, PRMT2, CD200R1, NRIP1, DGKZ, RASA1, H3F3B, A830073O21RIK
LAG3+ ANERGIC MOUSE CD4 (12)	https://www.ncbi.nlm.nih.gov/pubmed/19666526 (from analysis by (13))	1500004A13RIK, 1700019D03RIK, 2210010C04RIK, 2310001H17RIK, 2700008G24RIK, 2900026A02RIK, 4930452B06RIK, 4930506M07RIK, 5330417C22RIK, 5430401H09RIK, 6330403K07RIK, 6720475J19RIK, 8030451A03RIK, 9630010G10RIK, ABCB10, ABHD4, ACVR1, ADCK3, ADRBK2, AGR3, AHSP, AKR1B8, ALAS2, ALCAM, ALDH1A1, ALDH1A7, ALOX5AP, ANGPTL2, ANXA3, APOE, APOL11B, APOL8, AQP1, ARL6, ART3, ASCL2, ASNS, ATCAY, ATP2B2, ATRX, AURKB, B930095G15RIK, BC013712, BCL6, BLVRB, BMP2, BMPR2, BRCA1, BZRAP1, C2CD4B, C3, C730029A08RIK, CACNA1D, CAMK2N1, CAMP, CAPN5, CAR1, CAR2, CAR5B, CASC5, CASP3, CAV2, CC2D2A, CCDC112, CCDC28B, CCDC80, CCL1, CCL3, CCL6, CCL9, CCNB1, CCNE2, CCR8, CD109, CD200, CD24A, CD300LB, CD36, CD38, CD68, CD83, CD99L2, CDC6, CDCA8, CDKN2B, CEBPA, CEL, CELA1, CELA2A, CELA3B, CENPE, CENPK, CETN4, CHI3L3, CHN1, CHST2, CHTF18, CIT, CLDN13, CLEC4A3, CLEC4G, CLEC7A, CLIP3, CLPS, CLU, CNRIP1, COBLL1, COCH, COL4A1, COL4A2, CORO2B, CPA1, CPA2, CPB1, CREB3L2, CSDA, CSF1, CSF1R, CSF2RB, CSGALNACT1, CST7, CTGF, CTRB1, CTRL, CTSB, CTSH, CTTN, CX3CR1, CXCL10, CXCR7, CXXC5, CYBB, CYFIP1, CYP1A1, D10BWG1379E, D17H6S56E-5, D430019H16RIK, D630039A03RIK, DXERTD242E, DDC, DDX3Y, DGKI, DMBT1, DMXL2, DNAHC7B, DNASE1L3, DOCK4, DOCK7, DRAM1, DSP, DUSP14, DUSP16, DUSP4, DYNLT3, E130308A19RIK, EAR2, ECE1, EDN3, EEA1, EFNB2, EGR2, EIF2S3Y, EMILIN2, EMR4, ENPP2, ENTPD1, EOMES, EPB4.2, EPDR1, ERMAP, EVI5, EXPH5, F13A1, FAM132A, FAM20A, FAM43A, FAM55B, FAM55D, FAM59A,

		FAM81A, FAM84A, FARP1, FARP2, FBN2, FGD6, FHDC1, FIGNL1, FLRT2, FMNL2, FNDC3B, FPR2, FRMD4A, FZD6, GABARAPL1, GALM, GAS2L1, GATM, GCG, GDA, GJA1, GJB2, GLT28D2, GNA14, GNAQ, GP2, GPM6B, GPR116, GPR160, GPR35, GSTM1, GSTM5, GSTT3, GUCY1A3, GYPA, H2-AA, HAVCR2, HBB-B2, HCK, HEBP1, HEMGN, HIF1A, HIP1, HIVEP3, HJURP, HMBS, HMGN3, HP, ID2, IER5L, IFITM2, IFITM3, IFT122, IGDCC4, IGF1R, IGHM, IGHV14-2, IGSF6, IKZF2, IL10, IL1R2, IL21, ISPD, ITGB5, ITGB8, JAZF1, KDELC2, KDM5D, KEL, KIF13A, KIRREL3, KLF1, KLK1, KLRB1A, LAG3, LCN2, LIFR, LIMA1, LITAF, LMO2, LPAR3, LPL, LTF, LYN, LYZ1, LYZ2, MAF, MAGI3, MALT1, MAPRE2, 41341, MARCKSL1, MATN2, MET, MFSD2B, MGST1, MMD, MMP14, MPEG1, MPO, MT1, MTAP2, MTMR7, MYH10, MYH3, MYO1E, NA, NANOS1, NCAPG, NCRNA00086, NDN, NDRG4, NF2, NFATC1, NFE2, NFIL3, NGP, NHEDC2, NPAS4, NPNT, NR4A2, NRBP2, NRN1, NRP1, NT5E, NTF5, NTRK3, NUDT6, OCLN, ORC1, PADI4, PBK, PBX3, PDCD1, PDGFRL, PDZK1IP1, PDZRN3, PENK, PERP, PEX11A, PHACTR2, PISD-PS3, PKD2, PLAGL1, PLEK, PLEKHO1, PLOD2, PLSCR4, PLXDC2, PLXNB2, PNLIP, PNLIPRP1, PNLIPRP2, PPAP2A, PRELID2, PRKAR2B, PRKCA, PRNP, PRPF40A, PRSS2, PTGER2, PTGFRN, PTPLA, PTPN11, PTPN13, PTPN5, PTPRB, PTPRJ, PTPRS, PYGL, RAB39B, RALYL, RAPGEF5, RASSF6, RBM5, RBPJ, RCAN1, REEP1, REG1, RGS16, RHAG, RHD, RNASE1, RNF128, RPS6KC1, S100A10, S100A8, S100A9, S1PR3, SAMD11, SCCPDH, SCIN, SCLY, SDPR, SDR39U1, SEC16B, SEMA4C, SEMA7A, SERPINA9, SERPINC1, SERPINF1, SH3GL3, SH3RF1, SIRPB1A, SLC1A4, SLC22A15, SLC24A3, SLC25A13, SLC29A1, SLC35F5, SLC37A2, SLC38A5, SLC4A1, SLC5A3, SLC6A8, SLC7A10, SMC2, SMPDL3B, SNCA, SOSTDC1, SOX6, SPAG5, SPARC, SPIN4, SPIRE1, SPNA1, SPOCK2, SPP1, SRGAP3, SRXN1, STAB2, STC2, STFA3, STOM, STX11, SUSD2, SYNPO, SYT11, TAL1, TBC1D4, TCEAL8, TEX15, TFPI, TFRC, TG, TGFBI, TGM2, TIAM2, TIMP2, TJP1, TJP2, TM4SF1, TMBIM1, TMCC2, TMCC3, TMEM2, TMEM26, TMEM56, TMPRSS11E, TNFAIP2, TNFAIP8, TNFRSF9, TNFSF8, TNFSF9, TOX, TPMT, TREML4, TRFR2, TRIM10, TRIM12A, TRPS1, TSC1, TSHZ2, TSPAN2, TSPAN33, TSPAN8, TWSG1, UBR2, UHRF1, UNC13B, UTY, VAT1L, VDR, VSIG10, WIPF3, WISP1, ZBTB32, ZEB2, ZER1, ZFP612, ZG16

SUPPLEMENTAL FIGURE LEGENDS

Supplemental Figure 1. Characterization and baseline expression of TCR/CD3 complex in *de novo* HLA-A2-expressing Jurkat J76 CD8αβ T cells. (A) Representative histograms of surface markers on the parental Jurkat J76 CD8αβ cell line. Gray histograms correspond to unstained cells and colored histograms to cells stained with the indicated antibodies. **(B)** Representative histograms of HLA-A2 and HLA-A3 expression on codon-optimized A2-transduced J76 CD8αβ cells ($A2^{pos}/A3^{pos}$), parental J76 CD8αβ cells ($A2^{neg}/A3^{pos}$), T2 cells ($A2^{pos}/A3^{neg}$), and C1R cells ($A2^{neg}/A3^{pos}$). **(C)** Killing assays with NY-ESO-1-specific $A2^{neg}$ CD8 T cells (DMβ TCR variant) as effectors and indicated cells as targets, in the presence of increased concentrations of NY-ESO-$1_{157\text{-}165}$ native peptide. **(D)** Representative histograms of expression levels of the TCR/CD3 complex (panTCRαβ, Vβ13.1 and CD3ε) under steady-state culture conditions, in absence of antigen-specific stimulation. **(E)** Quantification of the expression of surface (i.e. extracellular) CD3ε and of total (i.e. intra- and extracellular) CD3ε. **(C and E)** Data are representative of 2 to 3 independent experiments.

Supplemental Figure 2. Phosphorylation levels of CD3ζ and ERK upon stimulation and basal expression levels of surface receptors in $A2^{pos}$ and $A2^{neg}$ J76 CD8αβ T cells. (A) Representative histograms of phosphorylation levels of CD3ζ and ERK1/2 after stimulation (with NY-ESO-1 multimer, OKT-3 or PMA/ionomycin) in $A2^{pos}$ (red histograms) and $A2^{neg}$ (blue histograms) J76 CD8αβ cells. **(B, C)** Representative histograms of CD5 and c-CBL expression **(B)** and of PD-1 and CD69 expression **(C)** in $A2^{pos}$ (red histograms) versus $A2^{neg}$ (blue histograms) J76 CD8αβ cells, under resting conditions.

Supplemental Figure 3. Basal expression levels of co-activating/inhibitory receptors on $A2^{pos}$ and $A2^{neg}$ primary CD8 T cells upon affinity-increased TCR transduction. (A) Representative histograms of the expression levels of co-activating/inhibitory receptors on $A2^{pos}$ (CRISPR/GFP) and $A2^{neg}$ (CRISPR/A2) primary CD8 T cells post-TCR transduction, in the absence of cognate antigen. Of note, no difference in any surface marker was observed between $A2^{pos}$ and $A2^{neg}$ untransduced CD8 T cells (gray histograms). **(B)** Relative expansion of $A2^{pos}$ (red lines) versus $A2^{neg}$ (blue lines) tumor-specific CD8 T cells upon TCR transduction, represented as NY-ESO-1^{pos} T cell fraction (upper panels) or as NY-ESO-1^{pos} variations from the initial transduction efficiency (bottom panels). Data are representative of 5 to 10 experiments.

Supplemental Figure 4. Surface staining and population doublings of primary A2^{pos} CD8 T cells with or without PD-1 blocking antibody. **(A)** Representative histograms of expression levels of co-activating/inhibitory receptors in A2^{pos} (CRISPR/A2) CD8 T cells post-TCR transduction, cultured over time with or without 20 µg/mL of nivolumab. **(B)** Population doublings of TCR-transduced A2^{pos} (CRISPR/A2) CD8 T cells cultured in the presence or the absence of 20 µg/mL nivolumab were assessed by periodic cell counting of living cells during 10 days and in the absence of cognate antigen. Data are representative of two independent experiments.

Supplemental Figure 5. Gene Set Enrichment Analysis (GSEA) of high-affinity versus optimal TCR variants. Genome-wide microarray analysis was previously performed on A2^{pos} primary CD8 T cells engineered with our panel of TCR variants against NY-ESO-1 (GSE42922) (2). **(A, B)** GSEA of available gene sets describing anergy (12), self-tolerance (10), and deletional tolerance (11) were found enriched in A2^{pos} CD8 T cells expressing **(A)** the high-affinity TCR wtc51m variant versus the wild-type one and **(B)** the high-affinity (i.e. TMα and wtc51m) relative to optimal-affinity TCR variants (i.e. G50A and DMβ), under steady-state culture conditions. Nominal p values and false discovery rates (FDR) are indicated for each gene set enrichment.

Supplemental Figure 6. CD107a degranulation and killing capacity of A2^{pos} and A2^{neg} primary CD8 T cells expressing affinity-increased TCRs. **(A)** Baseline expression levels of granzyme B and perforin in A2^{pos} and A2^{neg} primary CD8 T cells. **(B)** CD107a degranulation of A2^{pos} and A2^{neg} primary CD8 T cells upon stimulation with native NY-ESO-1 peptide-pulsed T2 cells at 0.1 µM. **(C)** Representative graphs (left panel) and quantification (right panel) of killing of Me275 cells pulsed with 1 µM of NY-ESO-1$_{157-165}$ native peptide at the indicated E:T ratios. **(D)** Representative graphs (left panel) and quantification of EC50 values (right panel) of killing of T2 cells pulsed with different concentrations of NY-ESO-1$_{157-165}$ native peptide (pp), at an E:T ratio of 10:1 by chromium release assays. **(A-D)** Data are means ± SD of 4-6 independent experiments.

Supplemental Figure 7. Expression levels of PD-1 and CD69 in A2^{neg} J76 CD8αβ cells during 14 days of co-culture with NA8 tumor cells. **(A)** Schematic representation of the experimental design; A2^{neg} J76 CD8αβ T cells of increased-affinity TCRs were co-cultured with either A2^{pos} or A2^{neg} NA8 tumor cells for 14 days in the absence of cognate antigen. **(B)**

Expression levels of PD-1 and CD69 on A2^{neg} J76 CD8αβ cells were performed at day 2, 4, 7, 10 and 14 of co-culture with either A2^{pos} or A2^{neg} NA8 tumor cells. Data are means ± SD of two independent co-culture experiments (n = 10). Matched P values are by two-way ANOVA followed by Sidak's multiple comparisons test; * $P \leq 0.05$ and **** $P \leq 0.0001$.

Supplemental Figure 8. **Dynamics of A2^{pos} versus A2^{neg} redirected primary CD8 T cell sub-populations in co-cultures following TCR transduction. (A)** Schematic representation of the A2^{pos} (CRISPR/mock) and A2^{neg} (CRISPR/A2) CD8 T cell co-cultures at an initial ratio of 1:1. TCR-untransduced CD8 T cells are depicted as gray-filled circles. **(B)** Flow cytometry gating (left panels) and quantification of A2^{pos} and A2^{neg} sub-populations (right panels) at the indicated days following TCR transduction and according to the different TCR affinity variants.

REFERENCES

1. Ran FA, Hsu PD, Wright J, Agarwala V, Scott DA, and Zhang F. Genome engineering using the CRISPR-Cas9 system. *Nat Protoc.* 2013;8(11):2281-308.
2. Hebeisen M, Baitsch L, Presotto D, Baumgaertner P, Romero P, Michielin O, et al. SHP-1 phosphatase activity counteracts increased T cell receptor affinity. *J Clin Invest.* 2013;123(3):1044-56.
3. Irving M, Zoete V, Hebeisen M, Schmid D, Baumgartner P, Guillaume P, et al. Interplay between T cell receptor binding kinetics and the level of cognate peptide presented by major histocompatibility complexes governs CD8+ T cell responsiveness. *J Biol Chem.* 2012;287(27):23068-78.
4. Allard M, Couturaud B, Carretero-Iglesia L, Duong MN, Schmidt J, Monnot GC, et al. TCR-ligand dissociation rate is a robust and stable biomarker of CD8+ T cell potency. *JCI Insight.* 2017;2(14).
5. Derre L, Bruyninx M, Baumgaertner P, Ferber M, Schmid D, Leimgruber A, et al. Distinct sets of alphabeta TCRs confer similar recognition of tumor antigen NY-ESO-1157-165 by interacting with its central Met/Trp residues. *Proc Natl Acad Sci U S A.* 2008;105(39):15010-5.
6. Chen JL, Stewart-Jones G, Bossi G, Lissin NM, Wooldridge L, Choi EM, et al. Structural and kinetic basis for heightened immunogenicity of T cell vaccines. *J Exp Med.* 2005;201(8):1243-55.
7. Dunn SM, Rizkallah PJ, Baston E, Mahon T, Cameron B, Moysey R, et al. Directed evolution of human T cell receptor CDR2 residues by phage display dramatically enhances affinity for cognate peptide-MHC without increasing apparent cross-reactivity. *Protein Sci.* 2006;15(4):710-21.
8. Robbins PF, Li YF, El-Gamil M, Zhao Y, Wargo JA, Zheng Z, et al. Single and dual amino acid substitutions in TCR CDRs can enhance antigen-specific T cell functions. *J Immunol.* 2008;180(9):6116-31.
9. Hebeisen M, Schmidt J, Guillaume P, Baumgaertner P, Speiser DE, Luescher I, et al. Identification of Rare High-Avidity, Tumor-Reactive CD8+ T Cells by Monomeric TCR-Ligand Off-Rates Measurements on Living Cells. *Cancer Res.* 2015;75(10):1983-91.
10. Schietinger A, Delrow JJ, Basom RS, Blattman JN, and Greenberg PD. Rescued tolerant CD8 T cells are preprogrammed to reestablish the tolerant state. *Science.* 2012;335(6069):723-7.
11. Parish IA, Rao S, Smyth GK, Juelich T, Denyer GS, Davey GM, et al. The molecular signature of CD8+ T cells undergoing deletional tolerance. *Blood.* 2009;113(19):4575-85.
12. Okamura T, Fujio K, Shibuya M, Sumitomo S, Shoda H, Sakaguchi S, et al. CD4+CD25-LAG3+ regulatory T cells controlled by the transcription factor Egr-2. *Proc Natl Acad Sci U S A.* 2009;106(33):13974-9.
13. Martinez GJ, Pereira RM, Aijo T, Kim EY, Marangoni F, Pipkin ME, et al. The transcription factor NFAT promotes exhaustion of activated CD8(+) T cells. *Immunity.* 2015;42(2):265-78.

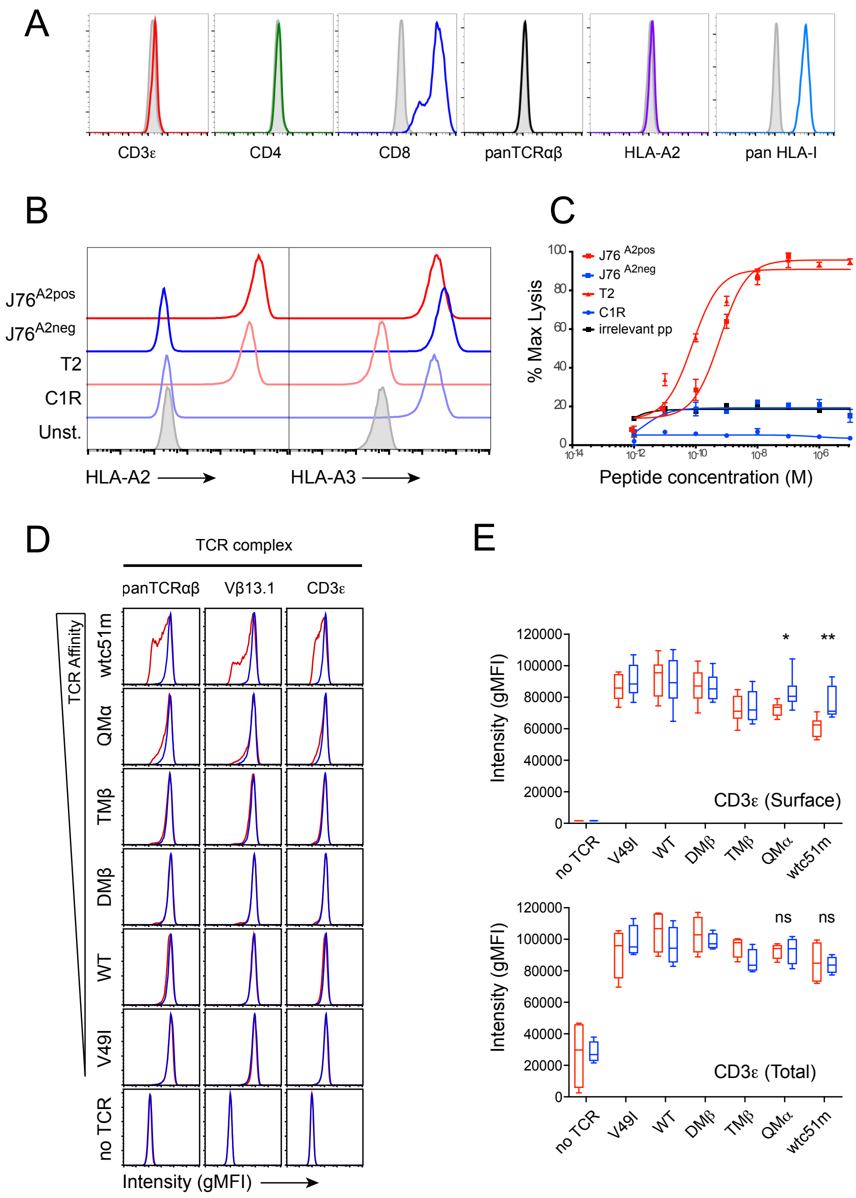

Supplementary FIGURE 1 - Duong et al.

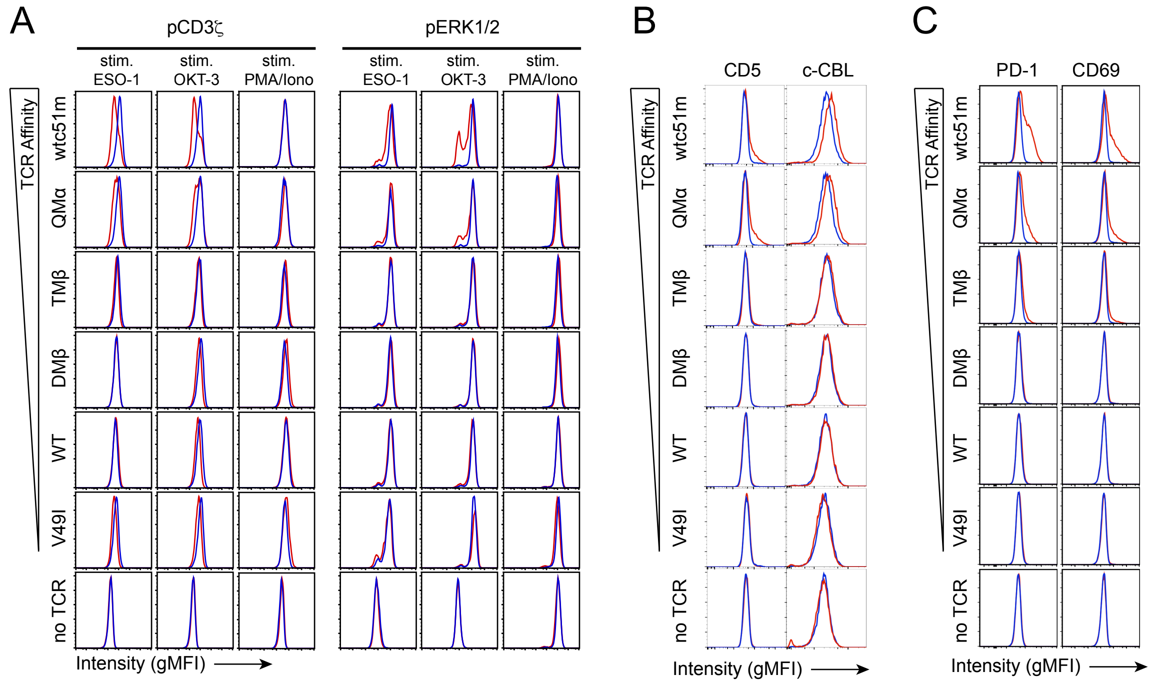

Supplementary FIGURE 2 - Duong et al.

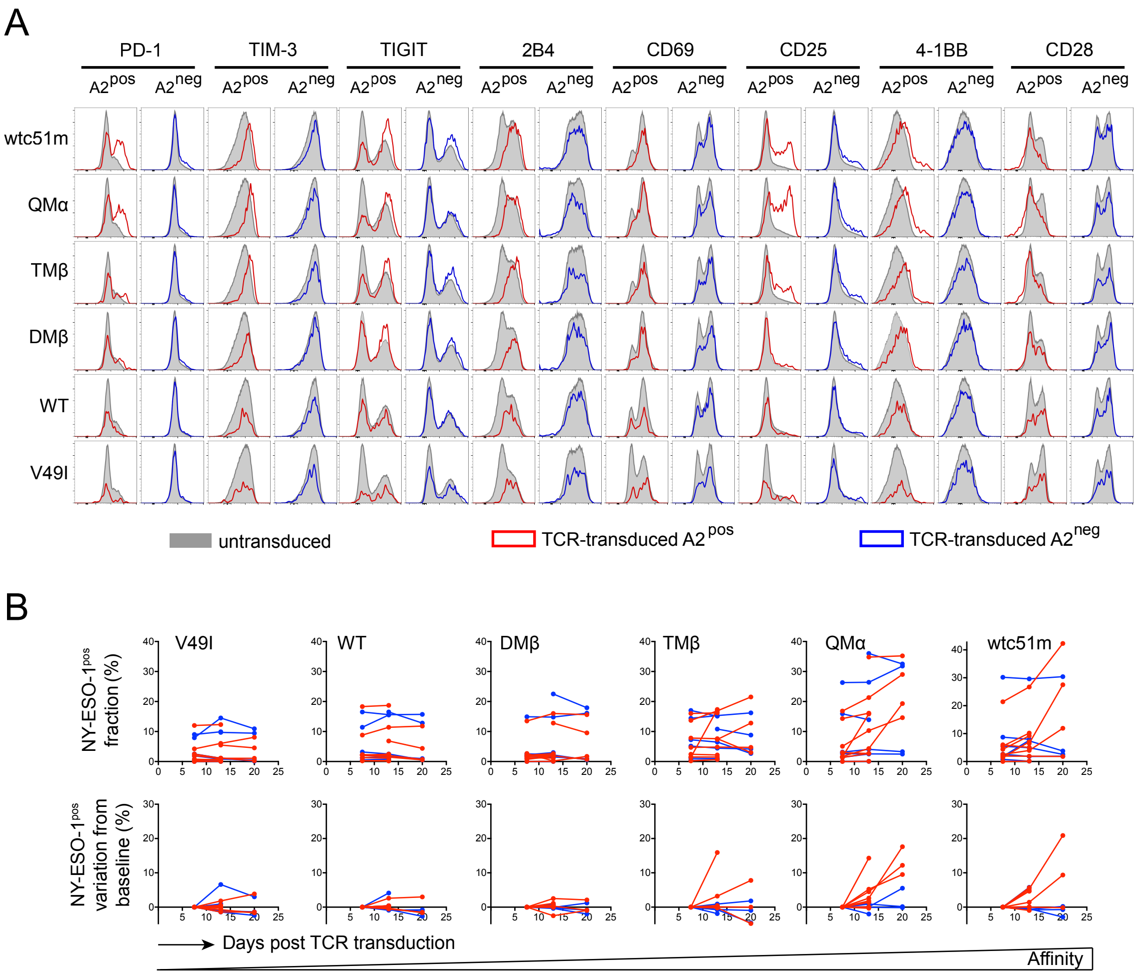

Supplementary FIGURE 3 - Duong et al.

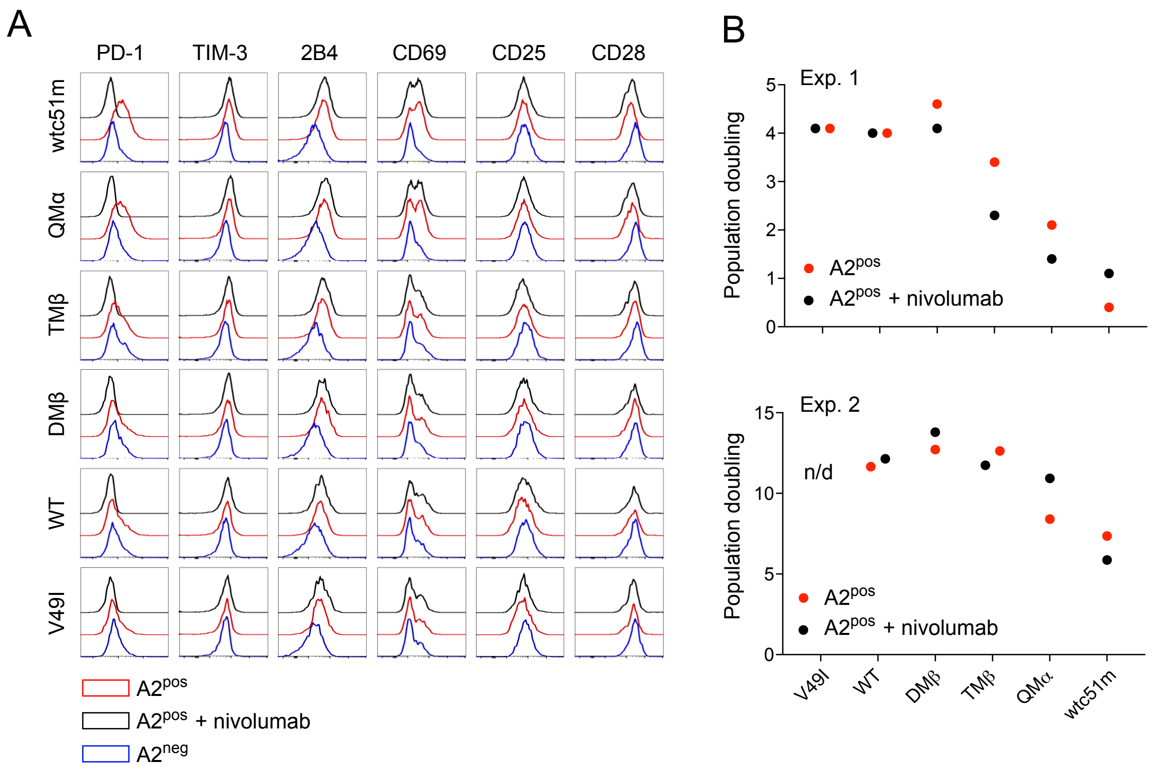

Supplementary FIGURE 4 - Duong et al.

Gene set enrichment analyses (GSEA) at baseline

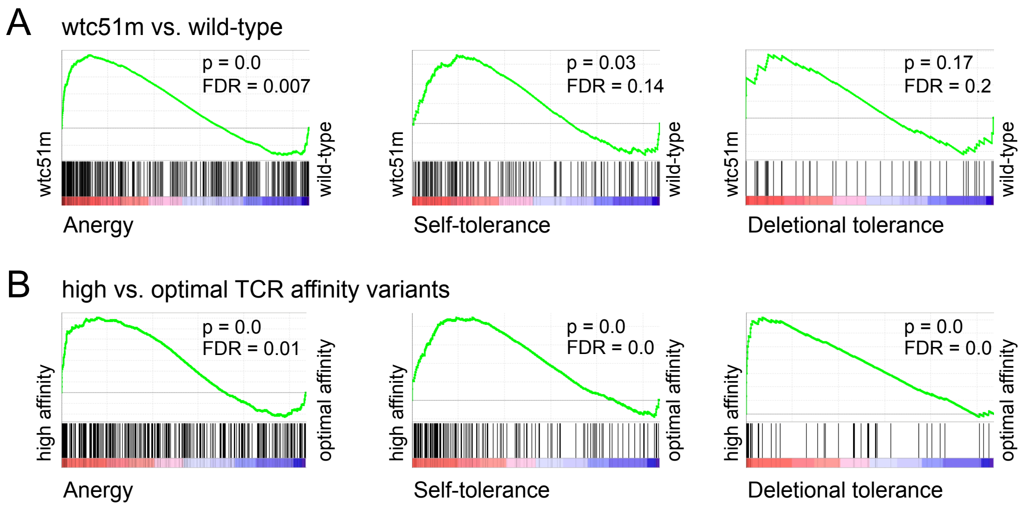

Supplementary FIGURE 5 - Duong et al.

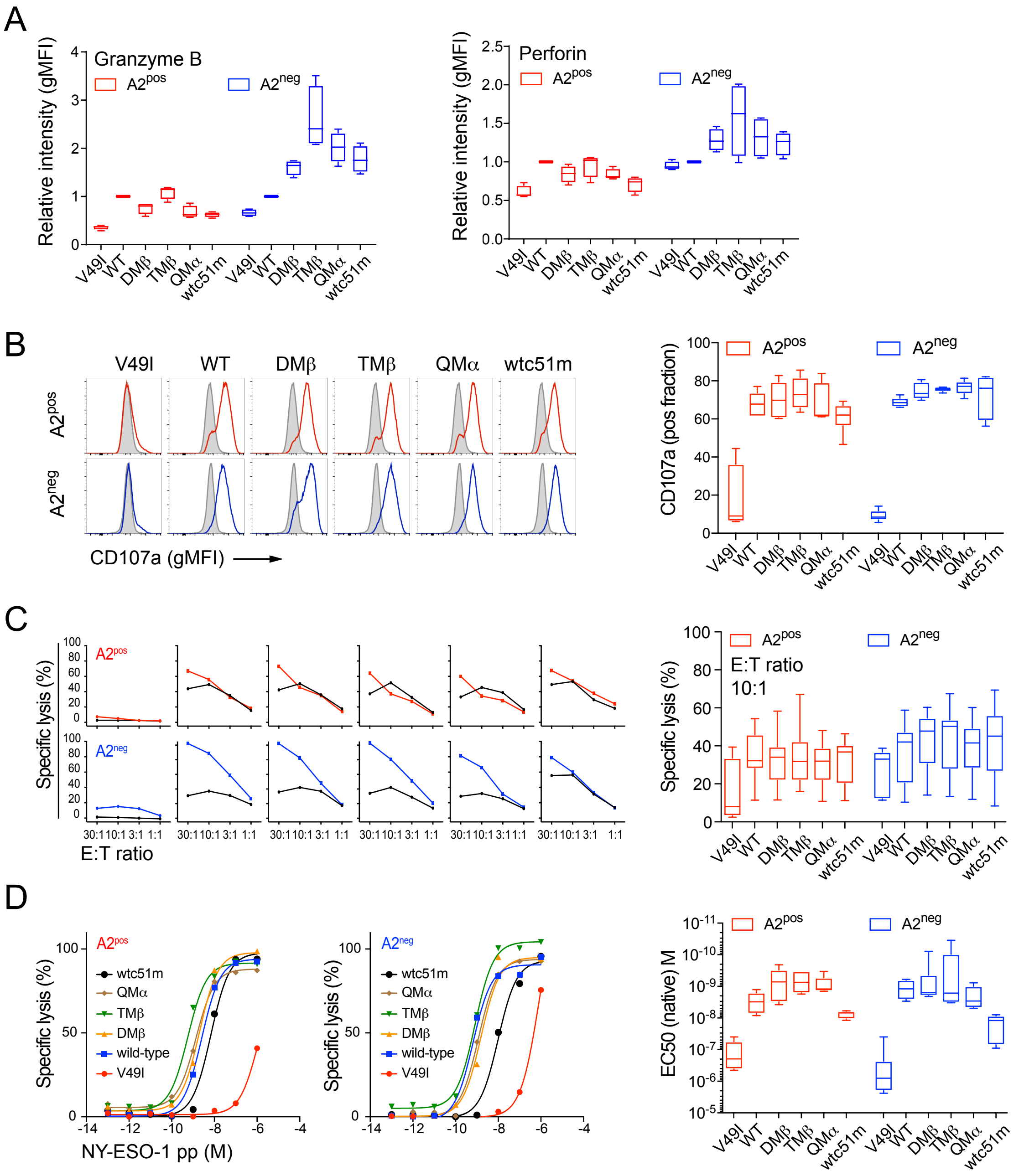

Supplementary FIGURE 6 - Duong et al.

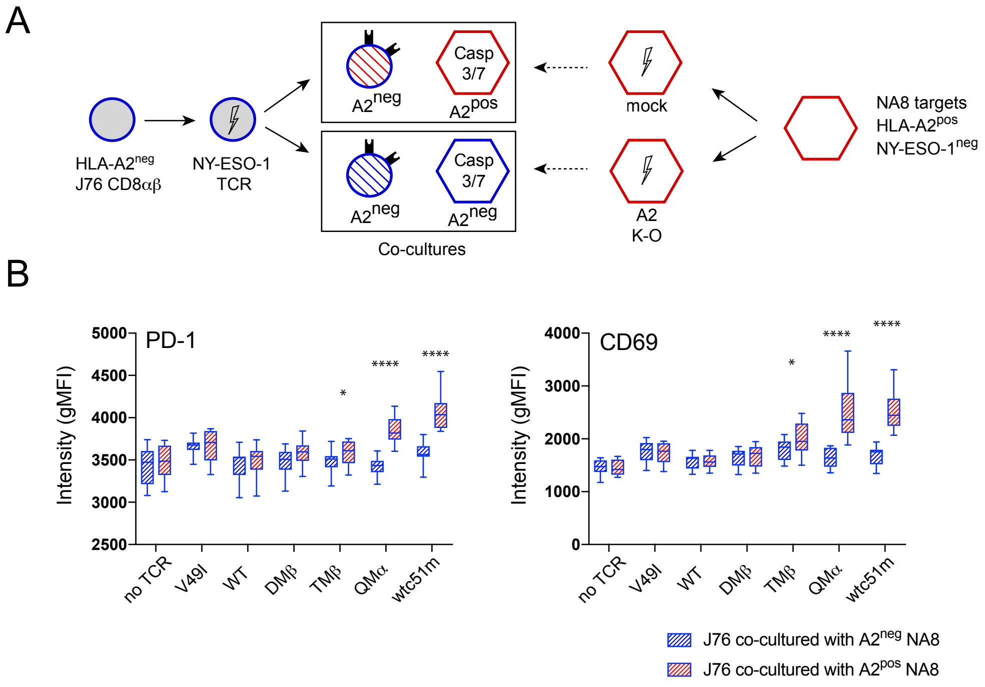

Supplementary FIGURE 7 - Duong et al.

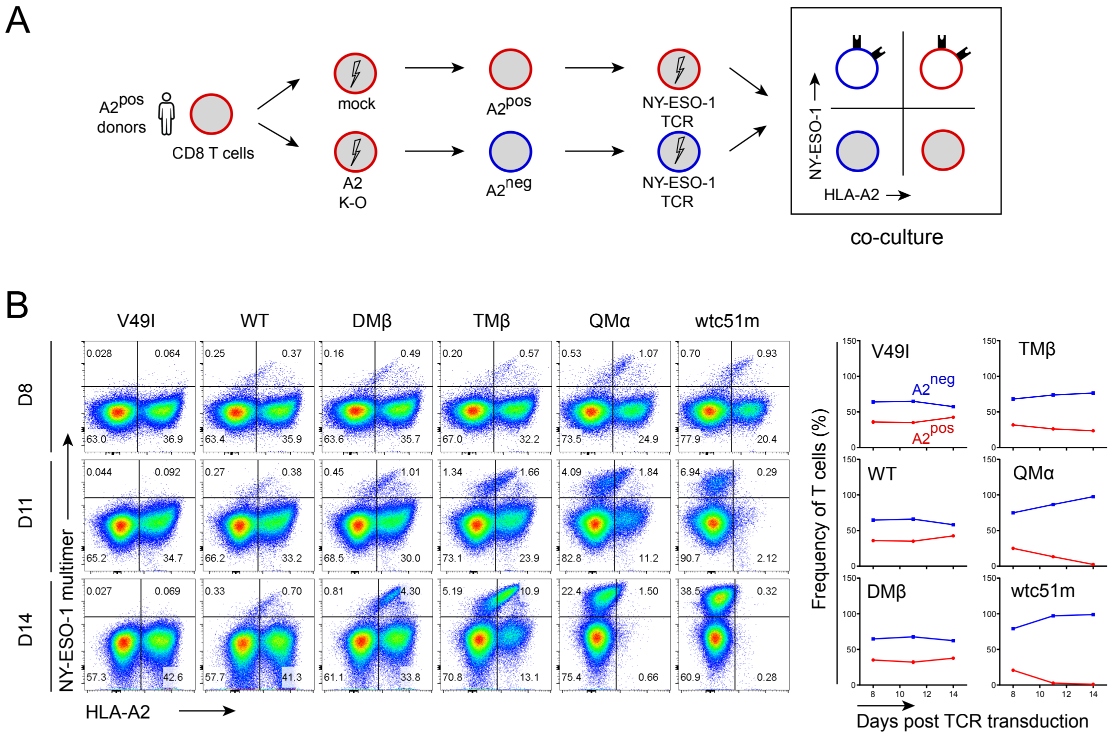

Supplementary FIGURE 8 - Duong et al.
